# Rational design of D(+)-biotin-conjugated resorcinol dibenzyl ethers as tumor-targeted PD-L1 inhibitors for precision cancer immunotherapy

**DOI:** 10.3389/fimmu.2026.1879839

**Published:** 2026-09-03

**Authors:** Jing-Jing Du, Rongling Liu, Lei Chen, Jian Zhang

**Affiliations:** 1 Hubei Provincial Key Laboratory of Occurrence and Intervention of Kidney Diseases, Hubei Provincial Engineering Research Center of Immunotherapy Drugs for Renal Tumors, Hubei Polytechnic University, School of Medicine, Huangshi, Hubei, China; 2 Huangshi Central Hospital, Huangshi, China

**Keywords:** D(+)−biotin, PD−1/PD−L1, precision cancer immunotherapy, small−molecule PD−L1 inhibitor, tumor−targeted

## Abstract

**Objectives:**

This study aims to discover and characterize novel D(+)-biotin-conjugated PD-L1 inhibitors for precision tumor-targeted cancer immunotherapy.

**Methods:**

Integrated strategies, including computer-aided molecular simulation, antiproliferative activity (CCK8) assay, surface plasmon resonance (SPR), pharmacokinetic evaluation, and *in vivo* antitumor efficacy assessment, were performed.

**Results:**

Compound SW-1 showed potent and selective inhibitory activity against PD-1/PD-L1 interaction with an IC_50_ of 5.6 nM, and high binding affinity to both human and murine PD-L1. Molecular docking and molecular dynamics simulations verified stable binding of SW-1 to the PD-L1 dimer interface with key intermolecular interactions. Pharmacokinetic studies in rats revealed favorable systemic exposure and a suitable half-life for *in vivo* application. In the B16-F10 melanoma mouse model, SW-1 achieved a more potent tumor growth inhibition (TGI = 64.9%) than the PD-L1 antibody (TGI = 44.2%), effectively promoted CD8^+^ T cell infiltration into the tumor microenvironment, and maintained favorable *in vivo* safety without obvious body weight loss.

**Conclusions:**

SW-1, as a highly potent biotin-conjugated PD-L1 small-molecule inhibitor, exhibits outstanding target activity, favorable pharmacokinetic properties, potent antitumor efficacy, and high safety. These results highlight SW-1 as a promising lead candidate for further development in precision tumor-targeted immunotherapy.

## 1 Introduction

Targeting the PD-1/PD-L1 immune checkpoint axis has become one of the most pivotal and rapidly evolving frontiers in modern anticancer drug discovery and cancer immunotherapy (1–3). Monoclonal antibody (mAb)-based PD-1/PD-L1 inhibitors have achieved significant clinical milestones across various solid tumors; however, their extensive therapeutic applicability remains constrained by several inherent pharmacological and safety limitations, including excessively prolonged systemic exposure (half-life often exceeding 2–3 weeks), modest objective response rates (typically 15–25% in unselected populations), and a notable occurrence of immune-related adverse events (irAEs) (4–6). Clinically important irAEs encompass immune-mediated colitis and, more critically, checkpoint inhibitor-associated myocarditis—a rare yet frequently fatal complication that considerably affects patient safety, treatment continuity, and long-term survival prospects (7, 8). In light of these limitations, small-molecule PD-L1 inhibitors have emerged as a compelling and urgently needed alternative, offering distinct pharmacological advantages: oral bioavailability, tunable pharmacokinetics (including shorter half-lives enabling rapid dose adjustment or discontinuation), and reduced risk of Fc-mediated effector functions—thereby mitigating off-target immune activation and improving therapeutic controllability (9–11).

**Figure 1:**
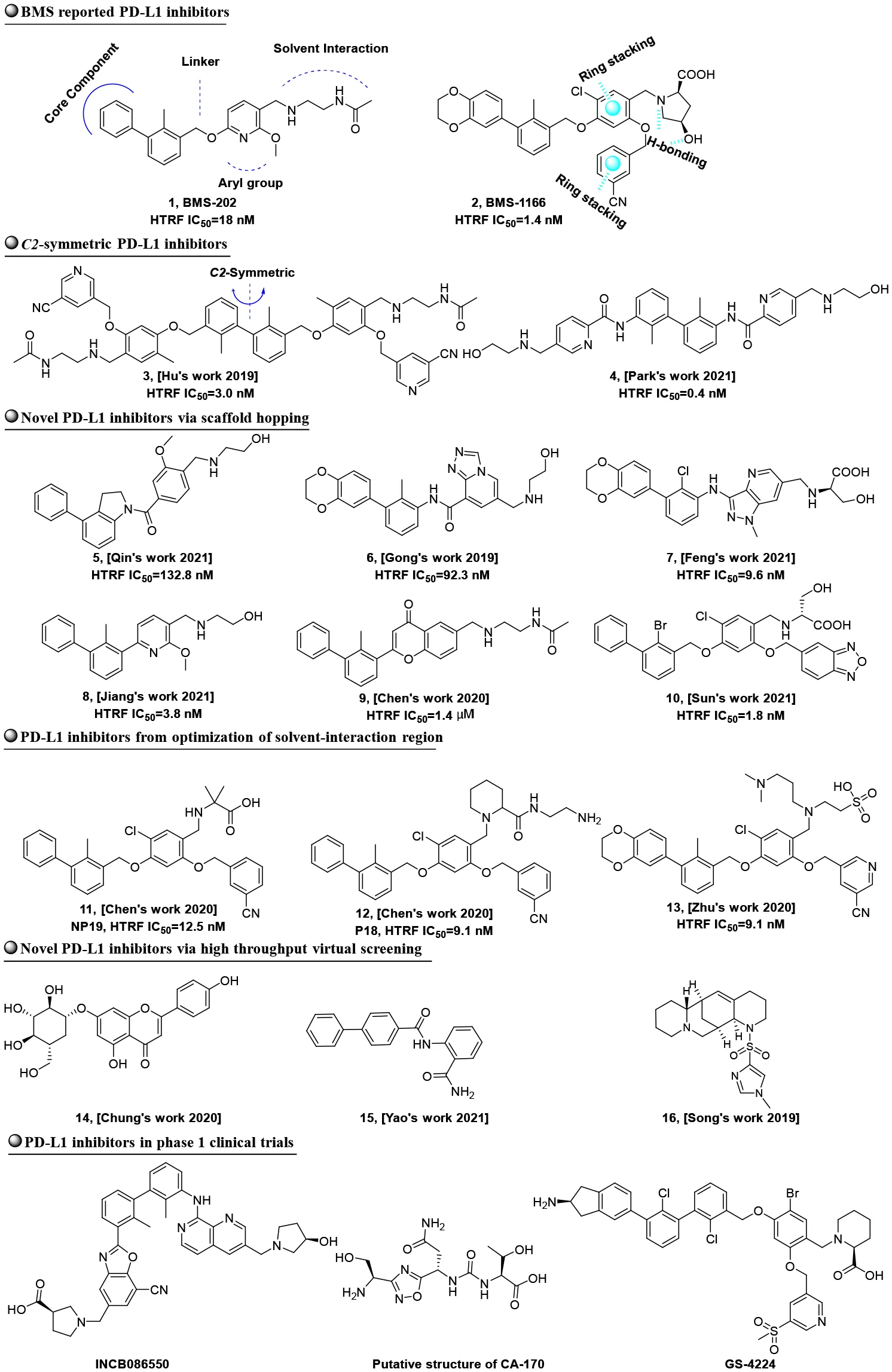
Small molecules with structural diversity targeting the PD-1/PD-L1 pathway. Reprinted (adapted) with permission from ref (18). Copyright {2023} American Chemical Society, license number 6277100092658.

To date, numerous structurally diverse small-molecule PD-L1 inhibitors have advanced into preclinical development and early-phase clinical trials (**Figure 1**) (12–14). Several non-antibody PD-L1 modulators are currently under clinical evaluation for solid tumors, including INCB086550 (NCT03762447, Incyte), CA-170 (NCT02812875, Aurigene Oncology), and GS-4224 (NCT04049617, Gilead Sciences). Despite this progress, a fundamental challenge persists: PD-L1 is constitutively expressed not only on tumor cells but also across diverse normal tissues—including lymphoid compartments (thymus, bone marrow, spleen, lymph nodes) and vital non-lymphoid organs (heart, lung, liver, kidney). Systemic, non-selective PD-L1 blockade inevitably disrupts peripheral immune homeostasis, leading to aberrant T-cell activation against self-antigens—a mechanism directly implicated in the onset, spectrum, and severity of irAEs (15–17). Thus, there remains a critical unmet need for PD-L1 inhibitors engineered for tumor-selective delivery, capable of preserving physiological PD-L1 function in healthy tissues while potently inhibiting its immunosuppressive activity within the tumor microenvironment (18).

**Figure 2:**
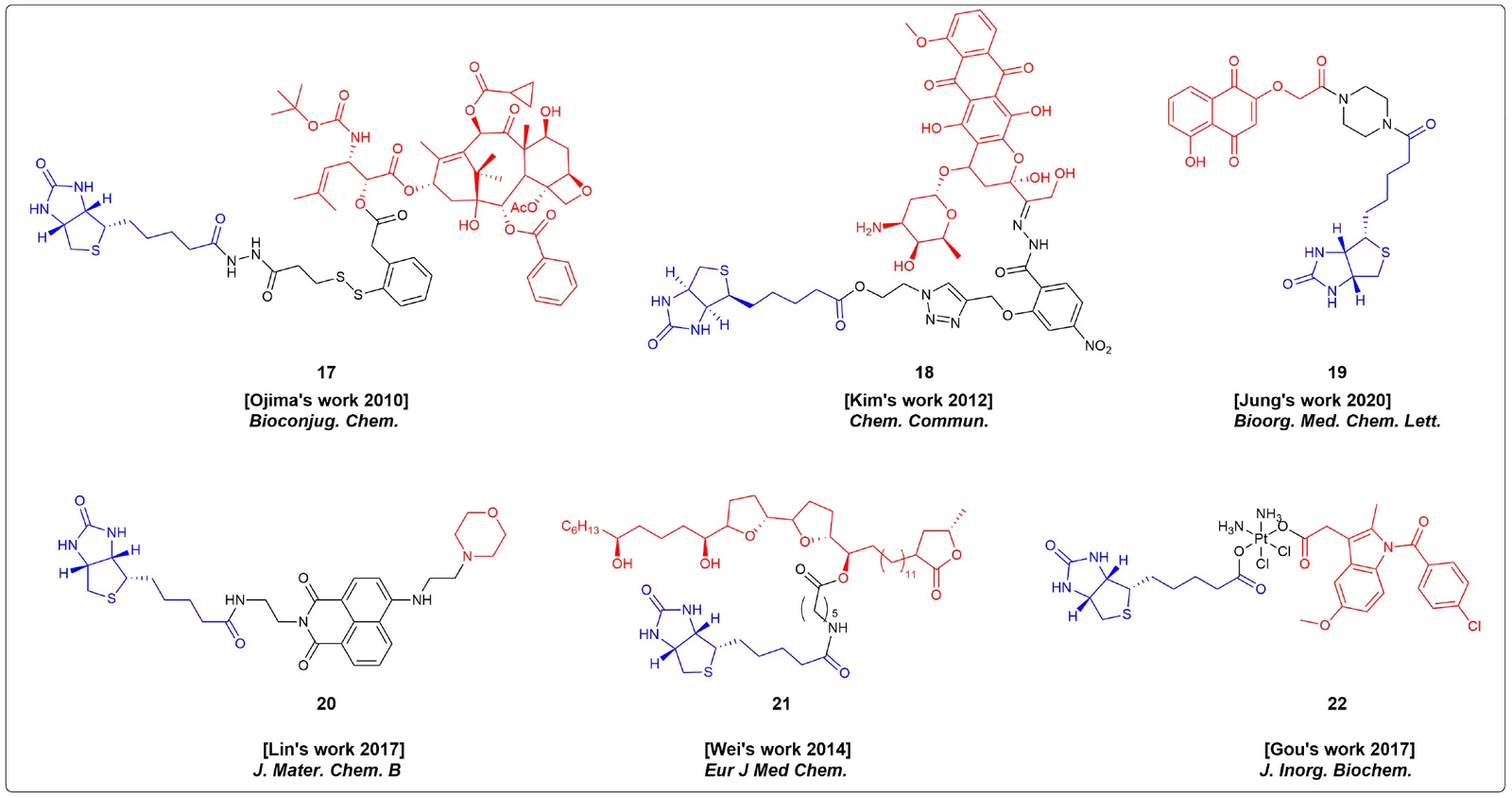
Small molecule agents that are modified with D(+)-biotin. Reprinted (adapted) with permission from ref (18). Copyright {2023} American Chemical Society, license number 6277100092658.

Accumulating evidence demonstrates that biotin receptors—particularly the sodium-dependent multivitamin transporter (SMVT)—are significantly overexpressed in multiple malignancies, including B16F10 melanoma, A549 lung adenocarcinoma, and MCF-7 breast cancer cells (19–21). This differential expression renders SMVT an attractive target for tumor-selective drug delivery. D(+)-Biotin, as a high-affinity endogenous ligand for SMVT, has been widely validated as a robust tumor-homing moiety. Conjugation of bioactive scaffolds to D(+)-biotin facilitates receptor-mediated uptake and intratumoral accumulation, markedly reducing off-target distribution and associated toxicity—aligning rigorously with the principles of precision oncology and spatially resolved immunotherapy (**Figure 2**). Importantly, emerging mechanistic studies further reveal that biotin itself modulates dendritic cell maturation and enhances CD8^+^ T-cell infiltration and cytotoxic function—indicating intrinsic immunostimulatory properties that synergize with PD-L1 blockade rather than merely serving as a passive targeting handle (18).

Our group has dedicated extensive efforts to developing small-molecule PD-L1 inhibitors. Building on structure–activity relationship (SAR) studies, we previously identified resorcinol dibenzyl ether derivatives as a privileged scaffold exhibiting potent PD-L1 binding affinity (sub-micromolar IC_50_), robust disruption of PD-1/PD-L1 protein–protein interaction, and significant antitumor efficacy in syngeneic mouse models. Beyond monovalent inhibition, we have also pioneered bifunctional modalities—including PD-L1-targeting PROTAC degraders and a combined strategy targeting both PD-L1 and DNA-PK—establishing versatile chemical platforms and translational proof of concept for next-generation immuno-oncology agents (22–24).

Motivated by the dual functionality of D(+)-biotin—as both a validated tumor-selective delivery vector and an endogenous immunomodulator—we herein report a rational molecular hybridization strategy to design and synthesize a novel class of D(+)-biotin-conjugated resorcinol dibenzyl ether inhibitors. This design strategically merges the high-affinity PD-L1 inhibitory core with the SMVT-targeting biotin motif to achieve spatiotemporal precision: (i) retaining uncompromised PD-L1 blockade potency at the tumor site, and (ii) restricting systemic PD-L1 engagement through preferential accumulation in SMVT-overexpressing tumor tissue. Collectively, this work introduces a functionally integrated, tumor-directed PD-L1 inhibitor prototype—one that simultaneously addresses efficacy, selectivity, and safety bottlenecks—and offers a generalizable paradigm for developing safer, smarter, and more effective immunotherapeutics.

## 2 Materials and methods

### 2.1 Chemical general methods

All reagents and solvents were purchased from commercial suppliers and used without further purification unless otherwise specified. BMS-202 (*InvivoChem*, Cat. No. V0770) was obtained as a positive control. Reaction progress was monitored by thin-layer chromatography (TLC), with visualization under UV light (254 nm) and/or iodine vapor staining. Column chromatography was performed on silica gel (300–400 mesh, Qingdao Haiyang Chemical Co., Ltd.) using gradient elution. ¹H and ¹³C NMR spectra were acquired on a Bruker AV-400 spectrometer (400 MHz for ¹H; 101 MHz for ¹³C), with chemical shifts reported in parts per million (ppm) relative to tetramethylsilane (TMS) as internal standard. High-resolution mass spectra (HRMS) were recorded on a Waters mass spectrometer in electrospray ionization (ESI) positive. Compound purity was assessed by high-performance liquid chromatography (HPLC) equipped with a C_18_ column (2.1 × 30 mm, 5 µm). Purity was determined from the UV chromatogram at 254 nm and confirmed to be ≥95% (area %) for all reported compounds.

### 2.2 Characterization and analytical test results of the target compounds

Compound SW-1 (*N*-(4’’-(((3-amino-2,2-dimethyl-3-oxopropyl)amino)methyl)-3’’-methoxy-2’-methyl-[1,1’:3’,1’’-terphenyl]-3-yl)-5-((3a*S*,4*S*,6a*R*)-2-oxohexahydro-1*H*-thieno[3,4-*d*]imidazol-4-yl)pentanamide) ^1^H NMR (400 MHz, *d*6-DMSO) δ 10.04 (s, 1H), 8.71 (s, 1H), 7.70 (s, 1H), 7.63 – 7.53 (m, 2H), 7.45 (d, *J* = 7.5 Hz, 1H), 7.35 (dd, *J* = 18.7, 8.3 Hz, 3H), 7.28 – 7.19 (m, 2H), 7.13 – 6.95 (m, 3H), 6.42 (d, *J* = 29.6 Hz, 2H), 4.31 (s, 1H), 4.12 (d, *J* = 26.4 Hz, 3H), 3.90 (s, 3H), 3.13 (s, 1H), 2.93 (s, 2H), 2.87 – 2.79 (m, 1H), 2.60 (s, 1H), 2.34 (t, *J* = 6.5 Hz, 2H), 2.09 (s, 3H), 1.70 – 1.37 (m, 6H), 1.21 (s, 6H). ^13^C NMR (101 MHz, *d*6-DMSO) δ 179.54, 171.76, 163.14, 157.72, 142.80, 142.47, 142.40, 139.66, 132.49, 131.25, 129.22, 129.09, 128.91, 126.02, 124.16, 121.57, 120.16, 118.03, 112.40, 61.46, 59.61, 56.09, 55.81, 48.01, 36.65, 28.65, 28.51, 25.51, 24.16, 18.92. HRMS m/z: calcd for C_36_H_46_N_5_O_4_S [M+H]^+^ 644.3271, found 644.3281. HPLC: *t*_R_ 12.72 min, purity 98.36%.

Compound SW-2 (*N*-(4’’-(((1,3-dihydroxy-2-(hydroxymethyl)propan-2-yl)amino)methyl)-3’’-methoxy-2’-methyl-[1,1’:3’,1’’-terphenyl]-3-yl)-5-((3a*S*,4*S*,6a*R*)-2-oxohexahydro-1*H*-thieno[3,4-*d*]imidazol-4-yl)pentanamide) ^1^H NMR (400 MHz, *d*6-DMSO) δ 10.02 (s, 1H), 7.70 (s, 1H), 7.55 (s, 1H), 7.48 (s, 1H), 7.40 – 7.30 (m, 2H), 7.22 (d, *J* = 6.6 Hz, 2H), 7.03 (dd, *J* = 18.8, 8.7 Hz, 3H), 6.42 (d, *J* = 29.3 Hz, 2H), 5.19 (s, 2H), 4.44 – 4.13 (m, 4H), 3.87 (d, *J* = 7.1 Hz, 3H), 3.64 (s, 6H), 3.16 (d, *J* = 20.3 Hz, 2H), 2.83 (s, 1H), 2.64 – 2.55 (m, 1H), 2.34 (d, *J* = 6.4 Hz, 2H), 2.09 (d, *J* = 7.0 Hz, 3H), 1.91 (d, *J* = 7.3 Hz, 1H), 1.67 – 1.34 (m, 6H). ^13^C NMR (101 MHz, *d*6-DMSO) δ 171.75, 163.15, 157.60, 142.80, 142.48, 139.64, 132.49, 129.18, 129.07, 128.92, 126.02, 124.18, 120.15, 118.04, 112.35, 61.46, 59.61, 58.59, 56.14, 55.81, 40.90, 36.65, 28.65, 28.51, 25.51, 21.48, 18.92. HRMS m/z: calcd for C_35_H_45_N_4_O_6_S [M+H]^+^ 649.3060, found 649.3032. HPLC: *t*_R_ 13.31 min, purity 96.23%.

Compound SW-3 (*N*-(4’’-((((*S*)-1-amino-3-hydroxy-1-oxopropan-2-yl)amino)methyl)-3’’-methoxy-2’-methyl-[1,1’:3’,1’’-terphenyl]-3-yl)-5-((3a*S*,4*S*,6a*R*)-2-oxohexahydro-1*H*-thieno[3,4-*d*]imidazol-4-yl)pentanamide) ^1^H NMR (400 MHz, *d*6-DMSO) δ 10.01 (s, 1H), 7.67 (s, 2H), 7.56 (d, *J* = 7.9 Hz, 1H), 7.46 – 7.31 (m, 4H), 7.21 (dd, *J* = 13.0, 7.5 Hz, 2H), 7.07 – 6.93 (m, 3H), 6.41 (d, *J* = 29.3 Hz, 2H), 5.21 (s, 1H), 4.32 (s, 1H), 4.15 (s, 1H), 3.92 (d, *J* = 5.4 Hz, 2H), 3.84 (s, 3H), 3.70 (s, 2H), 3.62 (s, 1H), 3.13 (s, 1H), 2.85 – 2.79 (m, 1H), 2.58 (d, *J* = 12.3 Hz, 1H), 2.33 (s, 2H), 2.08 (s, 3H), 1.53 (dd, *J* = 72.9, 27.2 Hz, 6H). ^13^C NMR (101 MHz, *d*6-DMSO) δ 171.82, 157.49, 142.73, 142.51, 139.57, 132.49, 130.43, 129.11, 128.94, 126.02, 124.22, 121.48, 120.18, 118.04, 112.19, 61.50, 59.65, 55.96, 55.80, 45.55, 36.65, 28.66, 28.51, 25.51, 18.95. HRMS m/z: calcd for C_34_H_42_N_5_O_5_S [M+H]^+^ 632.2907, found 632.2916. HPLC: *t*_R_ 13.02 min, purity 96.56%.

Compound SW-4 (*N*-(3’’-methoxy-2’-methyl-4’’-(((((*S*)-5-oxopyrrolidin-2-yl)methyl)amino)methyl)-[1,1’:3’,1’’-terphenyl]-3-yl)-5-((3a*S*,4*S*,6a*R*)-2-oxohexahydro-1*H*-thieno[3,4-*d*]imidazol-4-yl)pentanamide) ^1^H NMR (400 MHz, *d*6-DMSO) δ 9.99 (s, 1H), 7.67 (s, 2H), 7.55 (d, *J* = 7.9 Hz, 1H), 7.48 (d, *J* = 7.5 Hz, 1H), 7.34 (dd, *J* = 12.4, 7.4 Hz, 2H), 7.23 (d, *J* = 10.2 Hz, 2H), 7.02 (dd, *J* = 17.9, 8.6 Hz, 3H), 6.40 (d, *J* = 28.5 Hz, 2H), 4.32 (s, 1H), 4.07 (s, 3H), 3.88 (s, 4H), 3.13 (s, 1H), 2.95 (s, 1H), 2.83 (dd, *J* = 12.3, 3.6 Hz, 1H), 2.58 (d, *J* = 12.4 Hz, 1H), 2.34 (d, *J* = 6.3 Hz, 2H), 2.17 (s, 3H), 2.08 (s, 3H), 1.81 – 1.37 (m, 9H). ^13^C NMR (101 MHz, *d*6-DMSO) δ 177.20, 171.80, 157.48, 144.12, 142.77, 142.49, 142.43, 139.59, 132.48, 130.87, 129.11, 128.95, 126.04, 124.23, 121.54, 120.17, 118.05, 112.40, 61.49, 59.64, 56.14, 55.80, 53.14, 51.45, 46.72, 36.65, 29.72, 28.66, 28.51, 25.51, 24.80, 18.93. HRMS m/z: calcd for C_36_H_44_N_5_O_4_S [M+H]^+^ 642.3114, found 642.3126. HPLC: *t*_R_ 12.82 min, purity 97.11%.

Compound SW-5 (*N*-(4’’-(((1,3-dihydroxy-2-(hydroxymethyl)propan-2-yl)amino)methyl)-3’’-methoxy-2,2’-dimethyl-[1,1’:3’,1’’-terphenyl]-3-yl)-5-((3a*S*,4*S*,6a*R*)-2-oxohexahydro-1*H*-thieno[3,4-*d*]imidazol-4-yl)pentanamide) ^1^H NMR (400 MHz, *d*6-DMSO) δ 9.37 (s, 1H), 7.42 – 7.35 (m, 2H), 7.32 (t, *J* = 7.3 Hz, 1H), 7.27 – 7.21 (m, 2H), 7.07 (d, *J* = 7.1 Hz, 1H), 6.98 (d, *J* = 6.9 Hz, 1H), 6.91 (d, *J* = 9.8 Hz, 2H), 6.45 (s, 1H), 6.37 (s, 1H), 4.31 (s, 1H), 4.15 (s, 1H), 3.81 (d, *J* = 10.3 Hz, 6H), 3.44 (s, 4H), 3.14 (s, 2H), 2.83 (d, *J* = 11.9 Hz, 1H), 2.58 (d, *J* = 12.5 Hz, 1H), 2.36 (s, 2H), 1.99 – 1.86 (m, 10H), 1.58 (d, *J* = 46.4 Hz, 6H). ^13^C NMR (101 MHz, *d*6-DMSO) δ 172.57, 171.56, 163.26, 161.02, 157.09, 142.71, 142.50, 133.12, 130.34, 129.10, 128.47, 126.58, 125.95, 125.75, 121.57, 114.50, 111.81, 61.47, 61.19, 60.92, 59.60, 55.85, 55.80, 36.08, 28.73, 28.52, 25.73, 21.62, 18.13, 15.39. HRMS m/z: calcd for C_36_H_47_N_4_O_6_S [M+H]^+^ 663.3216, found 663.3231. HPLC: *t*_R_ 13.09 min, purity 97.29%.

Compound SW-6 (*N*-(4’’-(((3-amino-2,2-dimethyl-3-oxopropyl)amino)methyl)-3’’-methoxy-2,2’-dimethyl-[1,1’:3’,1’’-terphenyl]-3-yl)-5-((3a*S*,4*S*,6a*R*)-2-oxohexahydro-1*H*-thieno[3,4-*d*]imidazol-4-yl)pentanamide) ^1^H NMR (400 MHz, *d*6-DMSO) δ 9.37 (s, 1H), 8.11 (s, 1H), 7.59 (s, 1H), 7.46 (d, *J* = 6.6 Hz, 1H), 7.39 – 7.33 (m, 3H), 7.28 – 7.20 (m, 3H), 7.11 – 7.07 (m, 2H), 7.02 – 6.96 (m, 2H), 6.44 (s, 1H), 6.37 (s, 1H), 4.32 (s, 1H), 4.14 (s, 3H), 3.91 (s, 3H), 3.14 (s, 1H), 2.98 (s, 2H), 2.84 (d, *J* = 11.7 Hz, 2H), 2.60 (s, 1H), 2.36 (s, 2H), 1.93 (s, 2H), 1.90 (s, 2H), 1.64 – 1.40 (m, 6H), 1.22 (s, 6H). ^13^C NMR (101 MHz, *d*6-DMSO) δ 179.60, 178.38, 171.61, 163.17, 157.89, 142.62, 142.49, 142.00, 137.18, 133.02, 131.55, 130.49, 129.01, 126.60, 126.08, 125.91, 125.13, 121.76, 112.44, 61.54, 59.66, 56.20, 55.92, 47.98, 46.92, 36.06, 28.74, 28.60, 25.82, 24.15, 23.67, 18.17, 15.52. HRMS m/z: calcd for C_37_H_48_N_5_O_4_S [M+H]+ 658.3427, found 658.3431. HPLC: *t*_R_ 12.84 min, purity 97.11%.

Compound SW-7 (*N*-(4’’-((((*S*)-1-amino-3-hydroxy-1-oxopropan-2-yl)amino)methyl)-3’’-methoxy-2,2’-dimethyl-[1,1’:3’,1’’-terphenyl]-3-yl)-5-((3a*S*,4*S*,6a*R*)-2-oxohexahydro-1*H*-thieno[3,4-*d*]imidazol-4-yl)pentanamide) ^1^H NMR (400 MHz, *d*6-DMSO) δ 9.37 (s, 1H), 7.61 (s, 1H), 7.43 (d, *J* = 7.5 Hz, 1H), 7.39 – 7.32 (m, 2H), 7.30 – 7.19 (m, 2H), 7.08 (d, *J* = 7.4 Hz, 1H), 7.00 – 6.93 (m, 2H), 6.40 (d, *J* = 28.0 Hz, 2H), 4.32 (s, 1H), 4.16 (s, 1H), 3.86 (d, *J* = 20.2 Hz, 5H), 3.54 (s, 4H), 3.14 (s, 1H), 2.83 (d, *J* = 8.4 Hz, 1H), 2.58 (d, *J* = 12.3 Hz, 1H), 2.35 (s, 2H), 1.91 (d, *J* = 15.4 Hz, 6H), 1.64 (s, 2H), 1.46 (d, *J* = 45.0 Hz, 6H). ^13^C NMR (101 MHz, *d*6-DMSO) δ 175.06, 171.58, 167.42, 166.22, 163.45, 157.40, 146.41, 142.74, 142.36, 142.15, 132.85, 130.77, 130.54, 129.18, 128.78, 126.48, 126.09, 125.98, 124.99, 121.43, 112.13, 61.50, 59.63, 55.94, 55.85, 35.99, 28.68, 28.53, 25.76, 18.12, 15.44. HRMS m/z: calcd for C_35_H_44_N_5_O_5_S [M+H]+ 646.3063, found 646.3071. HPLC: *t*_R_ 12.90 min, purity 96.96%.

### 2.3 Molecular dynamics simulations

Structural files of target proteins were downloaded from the Protein Data Bank (PDB). We used AutoDock Vina to build protein-ligand complexes through molecular docking. All MD simulations were performed using GROMACS 2019 with the CHARMM36 force field. Each complex was embedded in a triclinic box, ensuring a 1.0 nm buffer zone between the solute and box walls. The system was solvated with TIP3P water, and counterions were added to neutralize charge and set ionic strength to 0.15 M, with periodic boundary conditions applied in all directions. The steepest-descent method was used to minimize energy, remove steric conflicts, and optimize initial structures. Two sequential equilibration processes were applied: a 100 ps NVT run at 310.15 K using the Berendsen thermostat, followed by a 100 ps NPT run at 1 bar controlled by the Berendsen barostat. The production simulation lasted 100 ns, with a 2 fs time step and the leap-frog integrator. Long-range electrostatic interactions were calculated with the PME method, van der Waals forces were characterized by the Lennard-Jones potential, and covalent bonds were restrained using LINCS. Trajectory data were analyzed by calculating RMSD, RMSF, Rg, SASA, hydrogen-bond number, and interaction energy, and by performing principal component analysis. The MM/PBSA method was finally utilized to estimate the protein-ligand binding free energy.

### 2.4 *In vitro* PD-1/PD-L1 binding assay

The recombinant PD-L1 protein was engineered to include a His affinity tag at its C-terminus. This tag enables specific binding to a monoclonal antibody targeting the 6His sequence, which is coupled to XL665 fluorescent labels. Correspondingly, the recombinant PD-1 protein was produced with a GST fusion tag. The paired detection antibody against GST was conjugated to europium cryptate as the signal reporter. In 384-well plates, 2 μL serially diluted compounds, 4 μL His-PD-L1, and 4 μL GST-PD-1 were sequentially added per well (total 10 μL). Then, 10 µL of pre-mixed anti-Tag1-Eu^3+^ and anti-Tag2-XL665 was added, the plate was sealed, and the plate was incubated for 2 hours at room temperature. Plates were incubated at room temperature in the dark for 120 min before dual-fluorescence detection at 665 nm (acceptor) and 620 nm (donor).

### 2.5 SPR analysis

All recombinant proteins, unless otherwise noted, were obtained from Sino Biological Inc.: murine PD-L1 (Cat. No. 50010-M08H) and human PD-L1 (Cat. No. 10084-HNAH). SPR imaging assays were performed using a PlexArray HT system. Briefly, hPD-L1 and mPD-L1 were each diluted to 0.5 mg/mL in sterile ultrapure water and covalently attached to bare, gold-plated PlexArray nanocapture sensor chips via amide coupling. Following this, serial dilutions of SW-1 analytes were injected into the flow cell (30 μL volume) at a steady flow rate using a non-pulsatile piston pump.

### 2.6 Antiproliferative activity assay

The antiproliferative activity of the target compounds was assessed *in vitro* against HepG2, B16-F10, and LO2 by the CCK-8 assay. Cells were seeded at 5000 cells per well in 96-well plates and cultured at 37 °C with 5% CO_2_ overnight. The medium was then replaced with 100 µL of fresh medium containing serial dilutions of the target compounds, and plates were incubated for 48 h. CCK-8 solution (10 µL) was added to each well, and the wells were incubated for an additional 1 h. Subsequently, absorbance was measured at 490 nm using a microplate reader. Finally, the growth-inhibitory effects, represented as IC_50_ values, were calculated.

### 2.7 Pharmacokinetic study in male Sprague-Dawley rats

This animal study was approved by the National Institutional Animal Care and Ethical Committee of Hubei Polytechnic University (Ethics Approval No. HBPU-IACUC-MED-2025-1520-1), and all operations complied with standard laboratory animal care specifications. Rats were raised under controlled conditions at 22–25 °C, 50%–60% humidity, and a 12 h light-dark cycle, with unlimited access to food and water. Before experiments, animals were acclimatized for at least one week. Food was withheld for 12 h before sampling, while the water supply was not restricted. Trained researchers collected blood from rat tail veins using gentle techniques to reduce pain, in accordance with the 3Rs principles. Experimental male Sprague-Dawley rats (250–260 g) were purchased from Liaoning Changsheng Biotechnology Co., Ltd. Compound SW-1 was given via three administration routes: oral gavage at 40 mg/kg, intravenous injection at 2 mg/kg, and intraperitoneal injection at 4 mg/kg. Then, 0.5 mL of blood was harvested at 0.0833, 0.25, 0.5, 1, 1.5, 2, 4, 6, 8, 12, 24, 36, and 48 h and stored in heparin-containing 1.5 mL polyethylene tubes. Sample detection was performed using an LC-MS/MS system consisting of a Shimadzu liquid chromatograph and an Applied Biosystems API 4000Q triple quadrupole mass spectrometer (18).

### 2.8 *In vivo* efficacy study in a melanoma model

This animal study was reviewed and approved by the National Institutional Animal Care and Ethical Committee (NIACEC) of Hubei Polytechnic University, under the ethical approval number HBPU-IACUC-MED-2025-1520-2. Animals were maintained under controlled laboratory conditions, with ambient temperature set at 22–25 °C, relative humidity maintained between 50–60%, and a standard 12-hour light/dark cycle. All mice had unrestricted access to sterilized standard chow and drinking water throughout the study. Upon study completion, mice were humanely euthanized via cervical dislocation performed by trained personnel. This method was specifically chosen to eliminate potential confounding effects of chemical euthanasia agents on downstream immunological and biochemical analyses. Death was confirmed by continuous cessation of both heartbeat and respiration before any tissue collection. Male C57BL/6 mice aged 6–8 weeks were procured from Liaoning Changsheng Biotechnology Co., Ltd. The total number of animals used was determined based on statistical power considerations and the experimental design, with strict adherence to the 3Rs (Replacement, Reduction, and Refinement) principles to minimize animal use and distress. Briefly, B16-F10 melanoma cells in logarithmic growth phase were harvested and resuspended in sterile PBS at a concentration of 2 × 10^6^ cells/mL. Each mouse received a subcutaneous inoculation of 100 µL of the cell suspension (containing 2 × 10^5^ viable B16-F10 cells) in the right flank, following established protocols for murine tumor transplantation models. When palpable tumors reached a volume of 50–100 mm³, mice were randomized into two groups: a vehicle control group (n = 6) and a treatment group (n = 6). SW-1 was formulated in a vehicle consisting of 30% PEG-300, 5% DMSO, and 65% normal saline. The vehicle control group received an equal volume of vehicle solution, while the treatment group received SW-1 at the specified dose via daily intraperitoneal injections for 15 days. The anti-mouse PD-L1 antibody (clone BE0101, BioXcell) was administered at a dose of 10 mg/kg every three days as a reference control. Tumor dimensions were measured using a calibrated electronic digital caliper, and tumor volume was calculated from the measured length and width. Tumor Growth Inhibition (TGI) was computed using the formula: TGI (%) = [1 − Wt/Wv] × 100%, where Wt and Wv represent the mean tumor weight of the treatment group and the vehicle control group, respectively (18).

### 2.9 Immunofluorescence of melanoma tumor tissues

Anti-CD8α monoclonal antibody was purchased from Abcam (catalog no. ab217344). For immunofluorescence staining, formalin-fixed paraffin-embedded (FFPE) tumor tissue sections were deparaffinized, rehydrated, and subjected to heat-induced epitope retrieval using citrate buffer (pH 6.0). Sections were then blocked with 5% normal goat serum in PBS for 1 h at room temperature, followed by overnight incubation at 4 °C with primary anti-CD8α antibody diluted 1:200 in antibody diluent. After thorough washing, Alexa Fluor 488–conjugated goat anti-rabbit IgG (H+L) secondary antibody was applied for 1 h at room temperature. Nuclei were counterstained with DAPI. Immunofluorescence signals were acquired using a laser-scanning confocal microscope, and CD8+ T-cell density and spatial distribution within the tumor parenchyma and invasive margin were quantified in ImageJ with standardized thresholding and region-of-interest annotation.

### 2.10 ELISA measurement of IFN-γ, TNF-α and IL-2 in mouse tumor tissues

The concentrations of IFN-γ, TNF-α and IL-2 in mouse tumor tissue homogenates were quantified using sandwich ELISA kits from Zhuocai Biotechnology (Shanghai, China). The catalog numbers were as follows: IFN-γ (Cat. No. ZC-37905), TNF-α (Cat. No. ZC-39024), and IL-2 (Cat. No. ZC-37976). Sample preparation: Tumour tissues were homogenized on ice in pre-cooled PBS supplemented with protease inhibitors at a mass-to-volume ratio of 1:9, then sonicated to ensure complete tissue lysis. The homogenate was centrifuged at 5000 × g for 10 min, and the supernatant was collected and stored at −80 °C. Repeated freeze-thaw cycles were strictly avoided prior to detection. ELISA procedure: All kits and tissue supernatants were equilibrated to room temperature before use. Briefly, 50 μL of standard solution or tissue sample was added to each well, followed by 100 μL of HRP-conjugated detection antibody (excluding blank wells), and the plates were incubated at 37 °C for 60 min. After incubation, the liquid was discarded, and each well was washed five times with 350 μL of washing buffer. Subsequently, 50 μL of substrate solution A and 50 μL of substrate solution B were sequentially added to each well, and the plates were incubated at 37 °C in the dark for 15 min to allow color development. The reaction was terminated by adding 50 μL of stop solution per well. The absorbance at 450 nm was measured within 15 min, and the sample cytokine concentrations were calculated using the standard curve.

## 3 Results

### 3.1 Rational design

**Figure 3:**
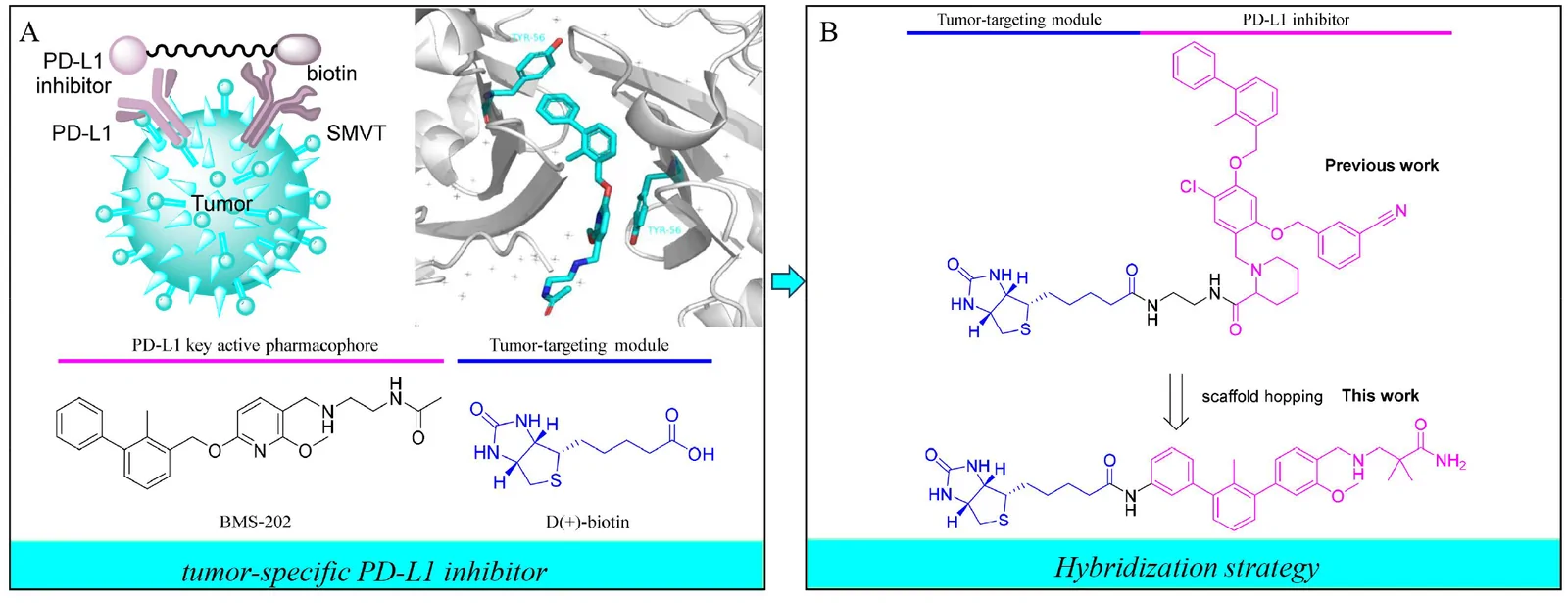
Rational design of biotin-conjugated tumor-targeted PD-L1 small-molecule inhibitors. **(A)** Schematic illustration of the dual-functional mode of action: the biotin-conjugated PD-L1 inhibitor selectively accumulates in tumor cells via the overexpressed SMVT transporter, while simultaneously blocking PD-L1 signaling to exert immunotherapeutic effects; **(B)** Molecular hybridization strategy for the design of biotin-tethered PD-L1 inhibitors, where the biotin-based tumor-targeting module is covalently linked to the core PD-L1 inhibitory pharmacophore derived from resorcinol dibenzyl ether.

To develop bifunctional, tumor-targeted PD-L1 inhibitors with improved drug-like properties, we employed a structure-guided rational design strategy, building on previously validated PD-L1 inhibitory scaffolds from the Chen group (16). As illustrated in **Figure 3A**, the core biphenyl pharmacophore of these inhibitors occupies the hydrophobic cleft formed by PD-L1 homodimerization and is stabilized by key interactions, including π-π stacking with Tyr56 and hydrogen-bonding networks, which are critical for disrupting PD-1/PD-L1 binding.

To confer tumor-specific targeting and reduce systemic immune-related adverse events, we leveraged the well-documented overexpression of SMVT on various cancer cells. As shown in **Figure 3B**, the previous bifunctional conjugate developed by the Chen group was constructed by directly linking the biotin moiety to a resorcinol dibenzyl ether-based PD-L1 inhibitor core. While this conjugate successfully combined PD-L1 inhibition with biotin-mediated tumor targeting, its relatively high molecular weight and complex substitution pattern presented challenges for further development, including suboptimal physicochemical properties, potential pharmacokinetic limitations, and synthetic complexity.

To address these issues and improve drug-likeness, we implemented a scaffold-hopping strategy to redesign the PD-L1 inhibitory module, aiming to reduce molecular weight while retaining key interactions with PD-L1. The resulting PD-L1 inhibitory core features a simplified biphenyl-fused benzoxazine scaffold that preserves the critical pharmacophore required for PD-L1 binding, including the hydrophobic biphenyl motif and hydrogen-bonding donors/acceptors. Compared to the original resorcinol dibenzyl ether scaffold from the Chen group, this optimized core exhibits a lower molecular weight, reduced structural complexity, and improved physicochemical properties, without compromising PD-L1 inhibitory activity.

Subsequently, we conjugated the biotin moiety (the tumor-targeting module, blue) to the redesigned PD-L1 inhibitory core (pink). The biotin moiety serves as an SMVT-targeting ligand, enabling selective enrichment of the conjugate in the tumor microenvironment, while the optimized PD-L1 inhibitory core retains full PD-L1 blockade activity. This rational design approach yields bifunctional molecules that simultaneously exhibit potent PD-L1 inhibition, tumor-specific targeting, and improved drug-like properties, thereby enhancing therapeutic selectivity, reducing off-target toxicities, and supporting further preclinical development.

### 3.2 Chemical synthesis

**Scheme 1:**
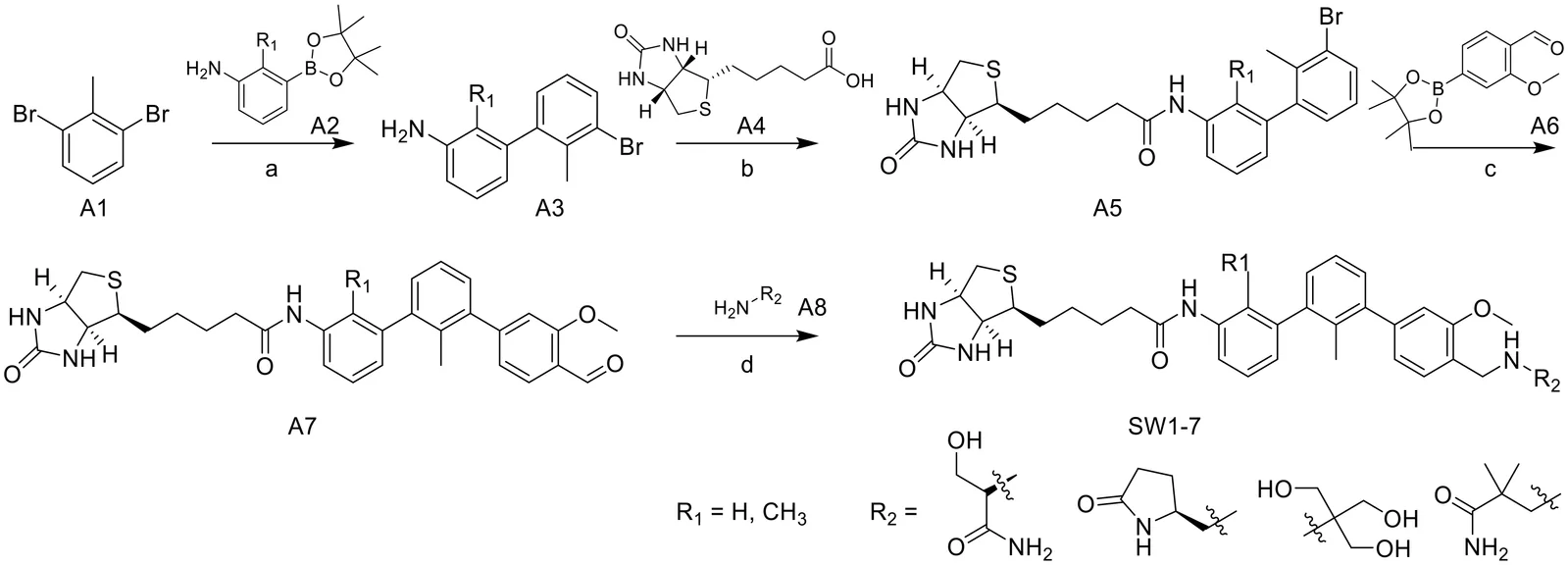
Synthesis of D(+)-Biotin-Conjugated PD-L1 inhibitors SW1-7 Reagents and conditions: **(a)** Pd(pph_3_)_4_, K_2_CO_3_, Dioxane-H_2_O, 80 °C, 10 h; **(b)** HATU, DIPEA, DMF, r.t, 2 h; **(c)** Pd(pph_3_)_4_, K_2_CO_3_, Dioxane-H_2_O, 80 °C, 10 h; **(d)** NaBH_3_CN, HOAc, DMF, 25 °C, 2 h.

The synthetic routes toward the target compounds SW1–7 are outlined in **Scheme 1**. Commercially available compound A1, 2,6-dibromotoluene, underwent a standard Suzuki coupling reaction with 2-aminoaryl boronic acid derivative A2 to give the biaryl intermediate A3 (2-methyl-[1,1’-biphenyl]-3-amine). Subsequently, intermediate A3 was subjected to an amide condensation reaction with biotin derivative A4 in the presence of HATU and DIPEA to afford the corresponding amide product A5. Next, A5 underwent a second Suzuki coupling reaction with 2-bromo-3-methoxybenzaldehyde-derived boronic ester to yield aldehyde intermediate A7. Finally, the target compounds SW1–7 were obtained via reductive amination between aldehyde intermediate A7 and amine derivatives A8 using sodium cyanoborohydride (NaBH_3_CN) in acetic acid. The identity and purity of the target compounds were rigorously confirmed by ¹H NMR spectroscopy; complementary characterization by ^13^C NMR, high-resolution mass spectrometry (HRMS), and HPLC demonstrated structural integrity and ≥95% chemical purity, meeting all predefined quality criteria for subsequent biological evaluation.

### 3.3 Biotin-conjugated tumor-targeted PD-L1 small-molecule inhibitors display potent target and cell inhibition

To establish the structure–activity relationship (SAR) of our biotin-conjugated PD-L1 inhibitors SW1–7, we systematically assessed how structural variations in the biotinylated tail and aromatic pharmacophore modulate inhibitory potency against PD-L1, quantified as IC_50_ values (**Table 1**), as well as their antiproliferative activity against murine melanoma (B16-F10), human hepatocellular carcinoma (HepG2), and normal liver (LO2) cell lines. The core biphenyl PD-L1-binding motif, conserved across all analogs, conferred consistent nanomolar-level PD-L1 inhibition, confirming the scaffold’s high target affinity. SW-1 emerged as the lead compound with optimal dual activity. The unconjugated parent PD-L1 scaffold, BMS-202, without biotin modification, was used as the control compound to clarify the functional role of biotin conjugation in target affinity and cellular activity. SW-1 potently inhibited PD-L1 with an IC_50_ of 5.6 ± 0.3 nM, whereas the untagged core inhibitor BMS-202 exhibited an IC_50_ of 61 ± 2.0 nM. SW-1 displayed robust antiproliferative activity against B16-F10 cells (IC_50_ = 0.5 ± 0.1 μM) while sparing normal LO2 cells (IC_50_ > 20 μM). Notably, SW-1 exhibited an IC_50_ of 2.4 ± 0.4 μM against HepG2 cells, further indicating its favorable therapeutic window among all synthesized analogs.

**Table 1:** *In vitro* PD-1/PD-L1 and antiproliferative activity of target compounds SW1-7^a^.

ID	Structure	Target IC_50_(nM)	Cell IC_50_(μM)		
		PD-L1	B16-F10	HepG2	LO2
SW-1	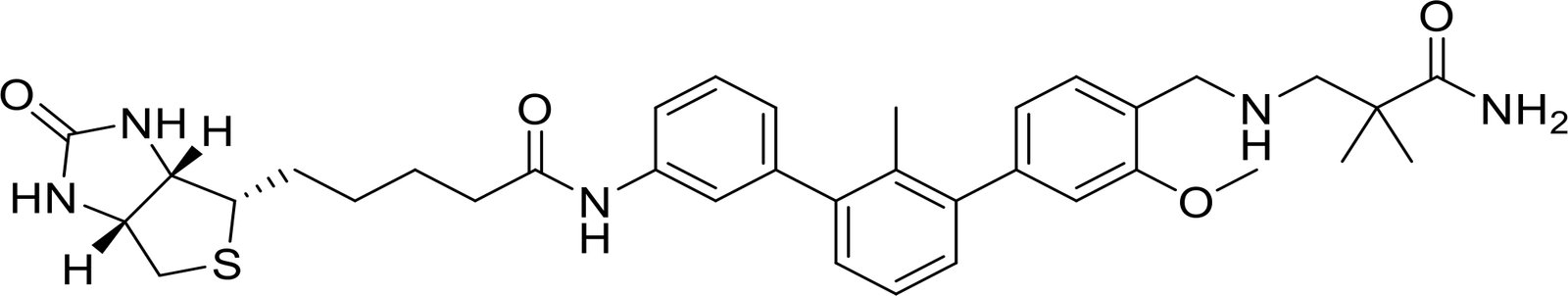	5.6 ± 0.3	0.5 ± 0.1	2.4 ± 0.4	>20
SW-2	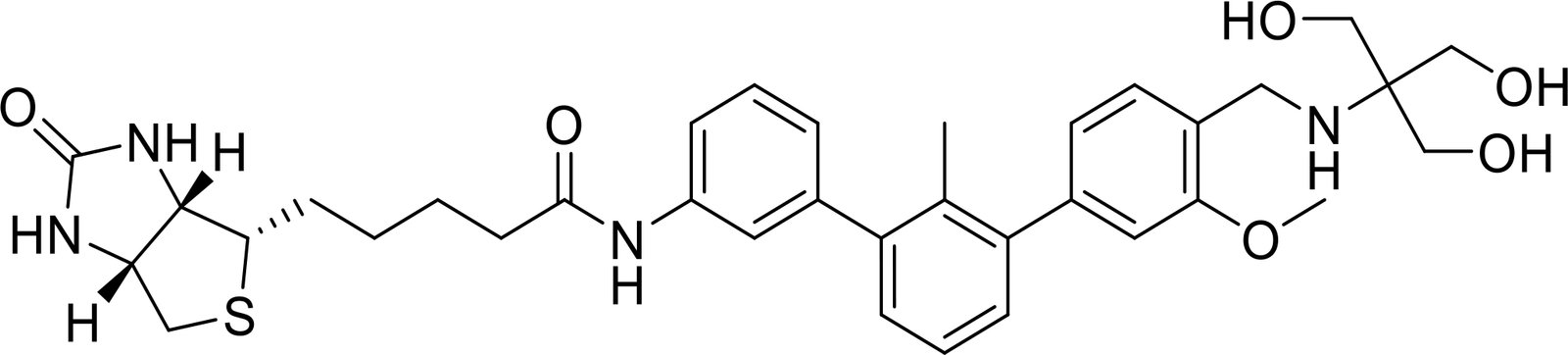	12.1 ± 1.1	4.2 ± 0.8	6.1 ± 0.5	>20
SW-3	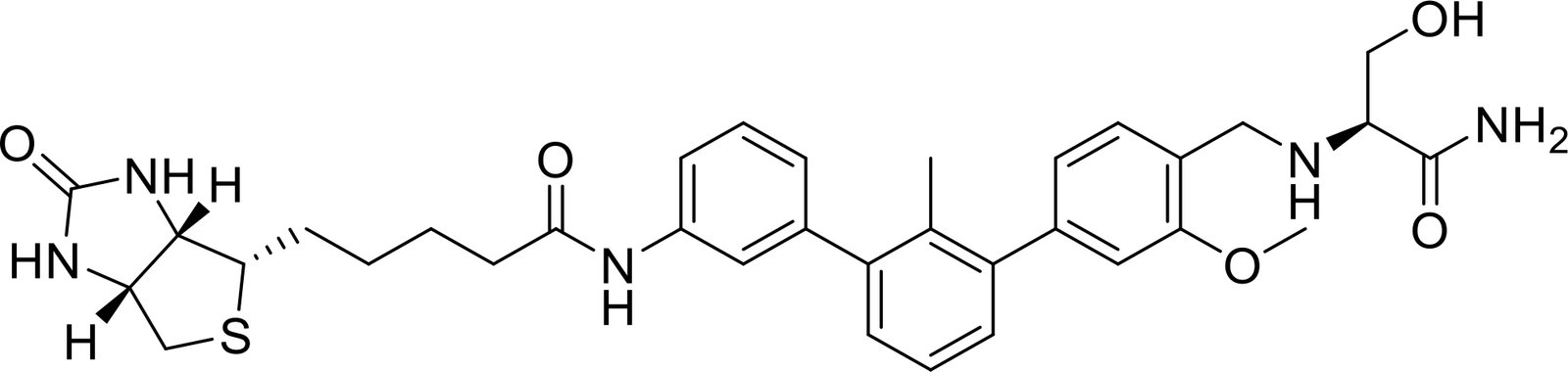	8.1 ± 0.5	9.6 ± 1.0	12.8 ± 2.2	>20
SW-4	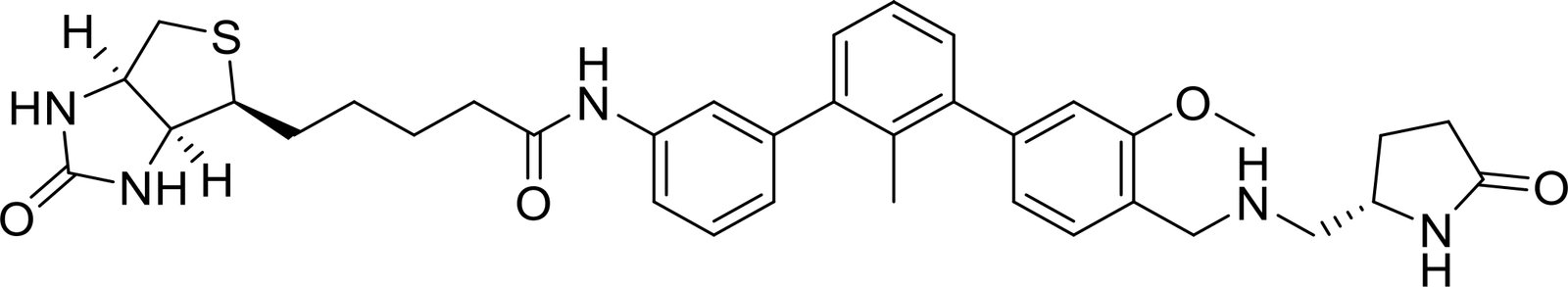	16.3 ± 0.9	12.3 ± 0.9	17.5 ± 1.7	>20
SW-5	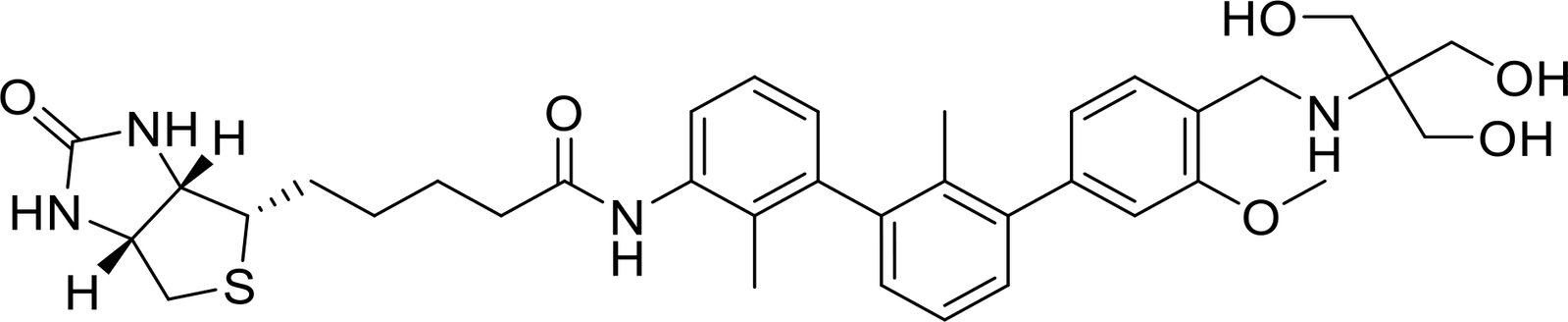	51.6 ± 2.1	>20	>20	>20
SW-6	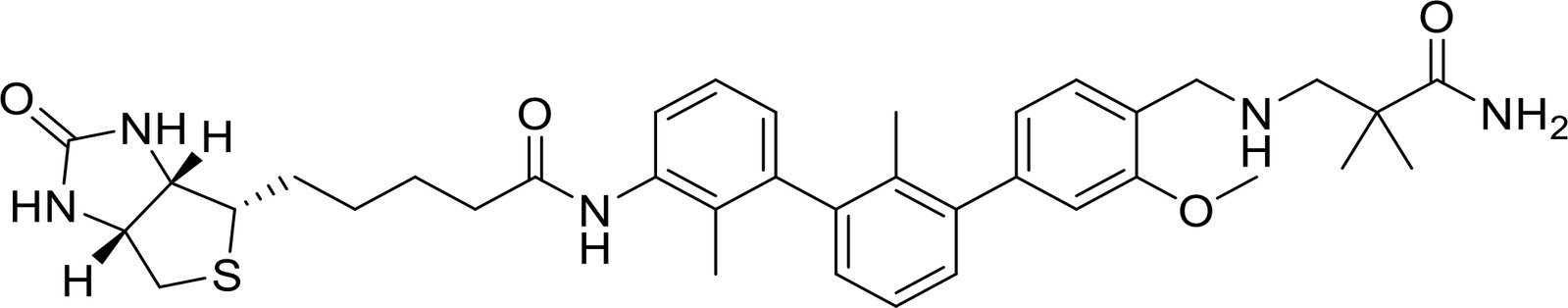	35.9 ± 2.3	12.6 ± 1.5	>20	>20
SW-7	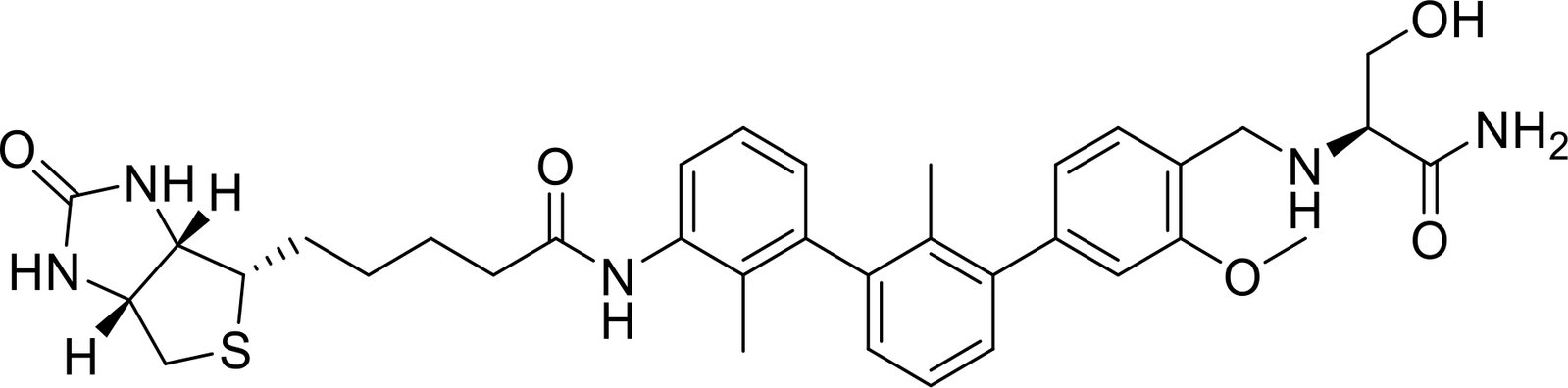	32.7 ± 1.8	>20	>20	>20
BMS-202	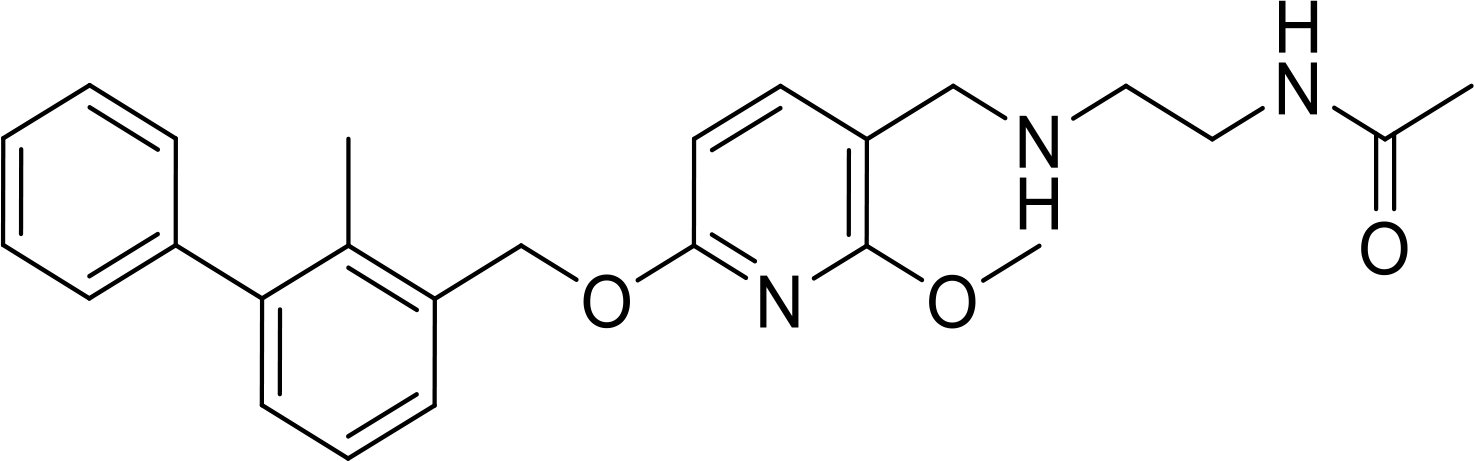	61 ± 2.0	>20	>20	>20

^a^ Cells were exposed to different concentrations of compounds continuously for 48 h before the determination of cell viability by the CCK-8 assay. IC_50_ values are presented as the mean ± standard error (SD).

Structure–activity analysis revealed key trends in both target inhibition and cellular activity. Modifications to the biotinylated tail directly influenced PD-L1 binding affinity; the unmodified primary amide derivative SW-1 showed the strongest activity, while substitution with polar groups or larger heterocyclic moieties progressively attenuated potency. SW-3 (hydroxamic acid, IC_50_ = 8.1 ± 0.5 nM) and SW-2 (triol derivative, IC_50_ = 12.1 ± 1.1 nM) retained sub-20 nM activity. In contrast, bulky cyclic substituents such as the succinimide moiety in SW-4 (IC_50_ = 16.3 ± 0.9 nM) or heterocyclic ring replacements like the benzoxazine group in SW-5 (IC_50_ = 51.6 ± 2.1 nM) significantly weakened binding, indicating that small, uncharged or weakly polar substituents are optimal for maintaining high PD-L1 affinity. A clear correlation was observed between PD-L1 inhibitory potency and cellular activity, with compounds exhibiting nanomolar PD-L1 IC_50_ values (SW-1 to SW-4) generally displaying low micromolar activity against B16-F10 cells, while analogs with reduced PD-L1 affinity (SW-5 to SW-7, IC_50_ > 30 nM) showed no significant cytotoxicity (IC_50_ > 20 μM), confirming that PD-L1 inhibition contributes to the observed cellular effects. HepG2 cells were less sensitive to these compounds overall, with only SW-1 and SW-2 displaying measurable activity (IC_50_ < 10 μM), likely reflecting cell-type-dependent differences in PD-L1 expression and dependency. Critically, all compounds showed no cytotoxicity toward LO2 normal liver cells (IC_50_ > 20 μM), demonstrating excellent selectivity for cancer cells over normal tissue, with SW-1 maintaining a particularly high therapeutic window of over 40-fold selectivity for B16-F10 cells relative to LO2 cells.

Collectively, these SAR findings highlight SW-1 as the optimal lead compound, combining the highest PD-L1 binding affinity with potent, selective antiproliferative activity while maintaining low toxicity to normal cells. The primary amide-substituted biotin tail and the unmodified biphenyl pharmacophore are critical for preserving both target affinity and cellular activity, making SW-1 the most promising analog for further biological evaluation.

### 3.4 Molecular modeling and molecular dynamics simulations of SW-1 with PD-L1

**Figure 4:**
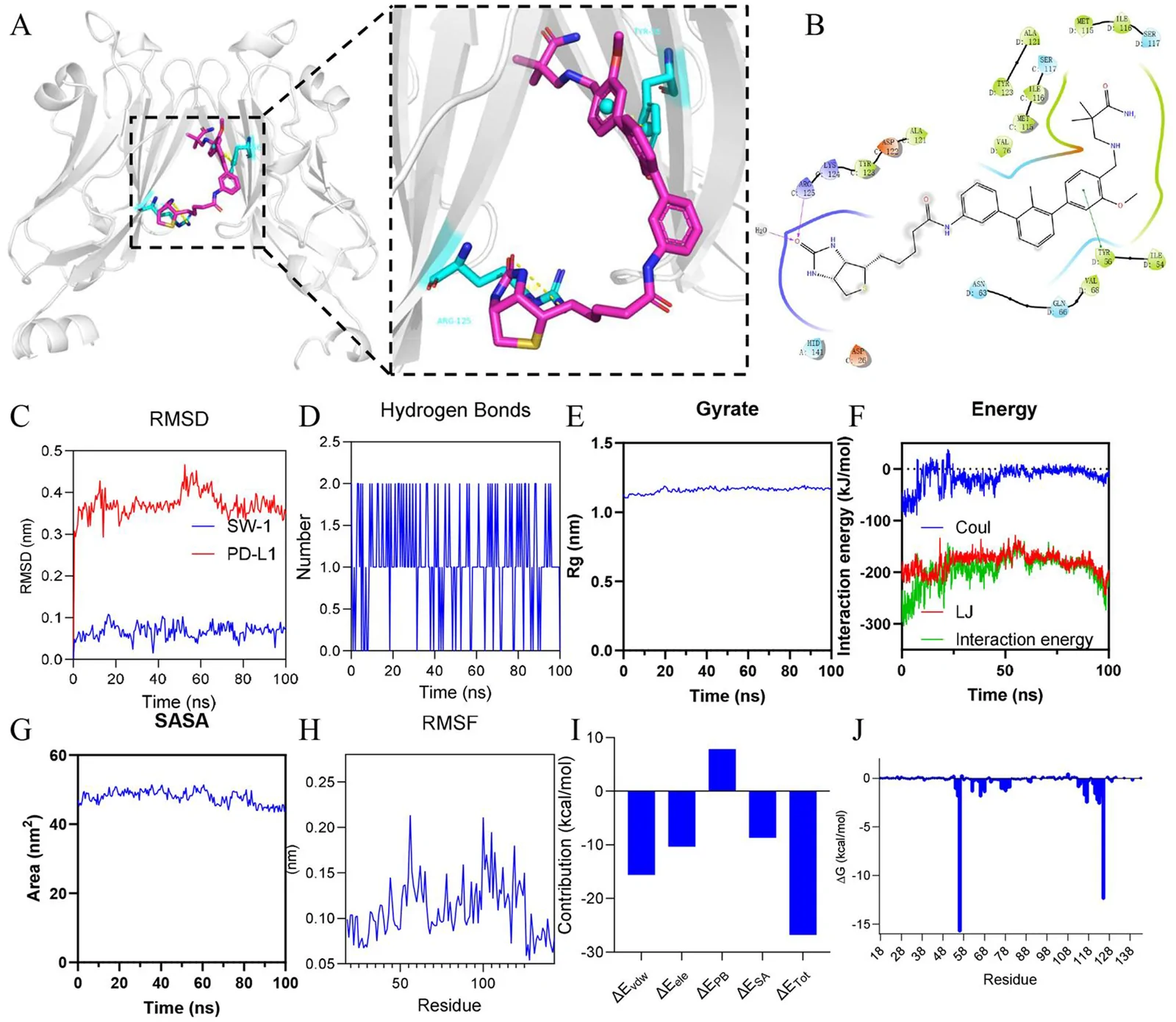
**(A)** Molecular docking model of SW-1 bound to the active site of PD-L1 (PDB ID: 5J89); **(B)** Predicted binding interactions between SW-1 and PD-L1 (PDB ID: 5J89) **(C)** RMSD of the PD-L1-ligand complex system during molecular dynamics simulations; **(D)** Hydrogen bond interactions between SW-1 and PD-L1 over the simulation period; **(E)** Radius of gyration (Gyrate) of PD-L1 in the PD-L1-SW-1 complex (reflecting protein conformational stability); **(F)** Interaction energy of the PD-L1-SW-1 complex; **(G)** SASA of the PD-L1-SW-1 complex; **(H)** RMSF of PD-L1 residues (indicating residue flexibility in the presence of SW-1); **(I)** MM/PBSA-based binding free energy calculation results of the PD-L1-SW-1 complex; **(J)** Total energy contribution of each PD-L1 residue to the binding interaction with SW-1.

To elucidate the molecular binding mode between SW-1 and PD-L1, molecular docking was performed using the PD-L1 crystal structure (PDB ID: 5J89) as the receptor model. Docking analysis demonstrated that SW-1 inserted deeply into the hydrophobic pocket at the PD-L1 dimer interface, forming a stable complex with key residues within the binding cleft. As shown in **Figure 4A**, the overall binding pose of SW-1 within the PD-L1 binding cleft was clearly displayed. The detailed interaction pattern revealed that the biphenyl fragment of SW-1 formed strong π–π stacking interactions with residues from both PD-L1 chains, accompanied by multiple hydrogen bonds between the biotin moiety and PD-L1 backbone or side-chain residues. The corresponding 2D interaction diagram (**Figure 4B**) further validated the tight spatial matching between SW-1 and PD-L1 and clarified the key noncovalent forces stabilizing the complex, including hydrogen bonds, hydrophobic interactions, and van der Waals contacts. These structural features are consistent with the potent biochemical activity of SW-1, as reflected by its low PD-L1 target IC_50_ value of 5.6 ± 0.3 nM.

To verify the dynamic stability of the SW-1–PD-L1 complex, a 100 ns molecular dynamics (MD) simulation was performed, and key structural parameters were analyzed. As depicted in **Figure 4C**, the root-mean-square deviation (RMSD) values of the PD-L1 dimer remained stable throughout the simulation, while the ligand SW-1 rapidly equilibrated and fluctuated within a narrow range after the initial stage. These results confirmed the complex’s high structural stability and suggested that SW-1 binding did not significantly distort the PD-L1 dimer. The dynamic hydrogen bond analysis (**Figure 4D**) showed that 1–2 hydrogen bonds were consistently maintained between SW-1 and PD-L1 throughout the simulation, supporting specific and sustained intermolecular recognition rather than nonspecific binding. Meanwhile, the radius of gyration (Rg) of the PD-L1 dimer remained narrowly distributed (**Figure 4E**), indicating that the overall folding and compactness of the protein were well preserved. As illustrated in **Figure 4F**, the interaction energy decomposition revealed that van der Waals (LJ) and electrostatic (Coul) energies contributed synergistically to binding, with the total interaction energy remaining highly negative throughout the simulation, further indicating a strong and stable binding profile. Similarly, the solvent-accessible surface area (SASA) remained stable (**Figure 4G**), suggesting limited exposure of hydrophobic regions and a favorable binding microenvironment. In addition, root-mean-square fluctuation (RMSF) analysis (**Figure 4H**) revealed that the binding interface residues exhibited markedly reduced flexibility, reflecting the rigidification effect induced by SW-1 binding. To quantitatively evaluate binding strength, the binding free energy (ΔG) contributions of key residues were analyzed (**Figure 4I**). Furthermore, per-residue energy decomposition (**Figure 4J**) revealed that critical residues provided dominant energy contributions, which were highly consistent with the docking results and highlighted their essential roles in maintaining the stability of the SW-1–PD-L1 complex. Taken together, these computational analyses provide reliable structural and dynamic evidence that directly rationalizes the potent PD-L1 inhibitory activity of SW-1 observed in biochemical assays. The stable binding pose, persistent intermolecular interactions, and favorable energy profile observed in silico are fully consistent with its nanomolar-level potency (IC_50_ = 5.6 ± 0.3 nM), confirming that SW-1 acts as a high-affinity PD-L1 inhibitor through well-defined molecular recognition mechanisms.

### 3.5 SW-1 demonstrated potent binding affinities to h/mPD-L1

**Figure 5:**
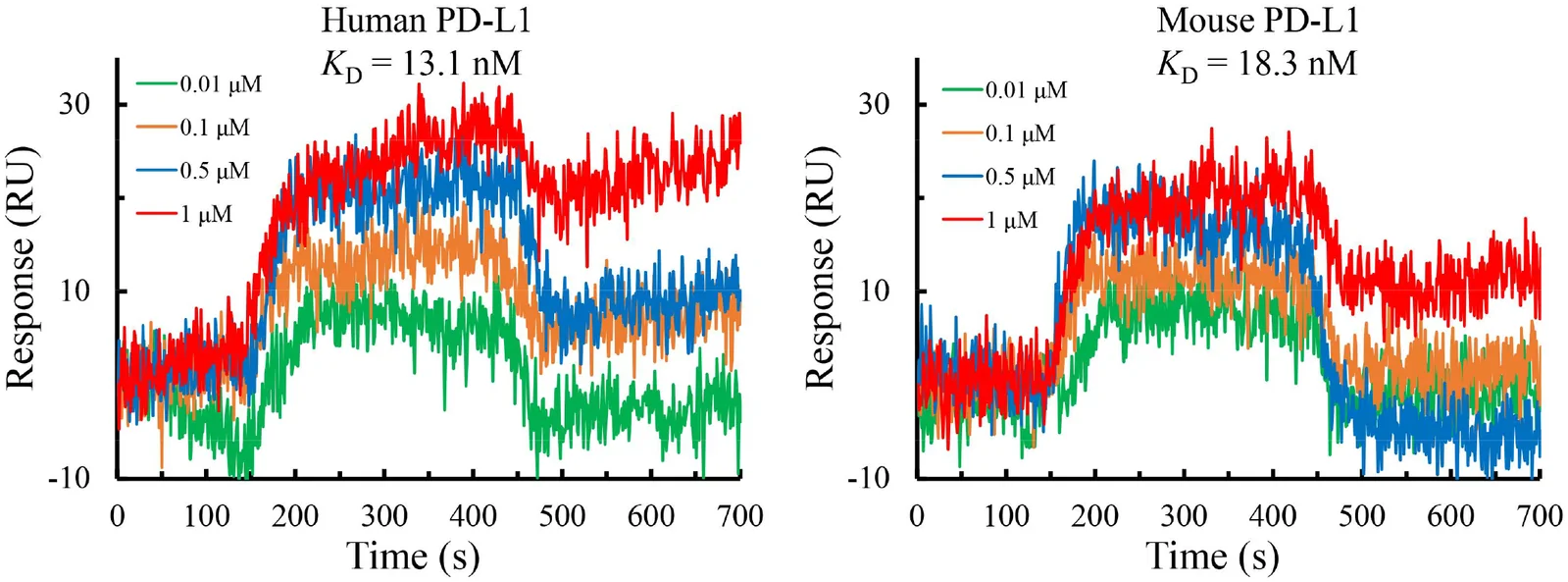
The binding affinity of SW-1 to h/mPD-L1 as determined by SPR experiments.

Having confirmed SW-1’s potent PD-L1 inhibitory activity in biochemical assays (IC_50_ = 5.6 ± 0.3 nM), we next directly quantified its binding affinity for both human and murine PD-L1 using surface plasmon resonance (SPR), an orthogonal label-free biophysical technique. As shown in **Figure 5**, the SPR sensorgrams demonstrate clear, concentration-dependent binding of SW-1 to both orthologs, with rapid association and slow dissociation kinetics across the tested concentration range (0.01–1 μM). Fitting of the steady-state responses yielded equilibrium dissociation constants (*K*_D_) of 13.1 nM for human PD-L1 and 18.3 nM for murine PD-L1. Both values fall within the low-nanomolar range, confirming that SW-1 engages PD-L1 orthologs with high and functionally relevant affinity. Notably, the measured *K*_D_ values are consistent with the sub-10 nM target IC_50_ observed in the biochemical PD-L1 inhibition assay. The slight discrepancy between the IC_50_ (5.6 nM) and SPR-derived *K*_D_ (13.1 nM) is expected, reflecting differences in assay formats: the inhibition assay measures functional blockade of the PD-1/PD-L1 interaction, while SPR directly quantifies binding affinity for the isolated PD-L1 protein. Together, these data establish a clear correlation between SW-1’s molecular binding affinity and its biochemical inhibitory potency. Importantly, SW-1 exhibited robust cross-reactivity with murine PD-L1 (*K*_D_ = 18.3 nM), indicating that its binding epitope is largely conserved between human and mouse orthologs. This species cross-reactivity validates the pharmacological relevance of SW-1 in immunocompetent mouse models, enabling *in vivo* evaluation of its PD-L1 blockade activity without species-related target incompatibility. Collectively, the SPR results provide orthogonal, quantitative evidence that SW-1 binds both human and murine PD-L1 with high affinity, directly supporting its observed nanomolar-level PD-L1 inhibitory activity and confirming its suitability for further biological and *in vivo* studies.

### 3.6 *In vivo* pharmacokinetic study

To support the *in vivo* efficacy evaluation of SW-1, we next characterized its pharmacokinetic (PK) profile in male Sprague-Dawley (SD) rats following intravenous (i.v.), intraperitoneal (i.p.), and oral (p.o.) administration (**Table 2**). Following a single i.v. dose of 2 mg/kg, SW-1 exhibited favorable systemic exposure with an AUC_(0–∞)_ of 1069.6 ± 116.8 ng/mL·h, a moderate volume of distribution (*V*z = 17.6 ± 3.2 L/kg), and a terminal elimination half-life (*t*_1_/_2_) of 6.4 ± 0.5 h. Clearance (CL) was low at 1.8 ± 0.2 L/h/kg, indicating slow systemic elimination. For the i.p. route (5 mg/kg), SW-1 showed rapid absorption, with a *T*_max_ of 0.25 h and a *C*_max_ of 996.6 ± 181.6 ng/mL. Systemic exposure was high, with an AUC_(0–∞)_ of 1377.7 ± 156.1 ng/mL·h, corresponding to an absolute bioavailability of ~51.6% relative to the i.v. dose. The elimination half-life remained stable at 6.6 ± 0.8 h, comparable to the i.v. group, confirming sustained systemic exposure. In contrast, oral administration (40 mg/kg) resulted in limited bioavailability: *C*_max_ was only 95.1 ± 25.9 ng/mL, with an AUC_(0–∞)_ of 654.1 ± 82.7 ng/mL·h, and an elimination half-life of 4.6 ± 1.8 h. The notably high CL (61.6 ± 7.8 L/h/kg) and large *V*z (402.8 ± 107.1 L/kg) suggest significant first-pass metabolism or poor gastrointestinal absorption, making the oral route unsuitable for *in vivo* efficacy studies.

**Table 2:** Primary pharmacokinetic properties of SW-1 in SD rats (n = 6, Mean ± SD).

Pharmacokinetic parameters	I.V. (SW-1, 2 mg/kg, n = 6)	I.P. (SW-1, 5 mg/kg, n = 6)	P.O. (SW-1, 40 mg/kg, n = 6)
AUC_(0–t)_ (ng/mL·h)	1059.7 ± 120.1	1269.9 ± 150.5	648.1 ± 74.9
AUC_(0–∞)_ (ng/mL·h)	1069.6 ± 116.8	1377.7 ± 156.1	654.1 ± 82.7
MRT_(0–t)_ (h)	7.7 ± 3.6	6.9 ± 1.3	8.3 ± 0.7
MRT_(0–∞)_ (h)	8.1 ± 3.4	7.2 ± 5.5	8.7 ± 0.3
*t*_1/2_ (h)	6.4 ± 0.5	6.6 ± 0.8	4.6 ± 1.8
*T*_max_ (h)	0.083	0.25	1.5 ± 0.7
CL (L/h/kg)	1.8 ± 0.2	3.5 ± 1.3	61.6 ± 7.8
*V*z (L/kg)	17.6 ± 3.2	29.7 ± 1.4	402.8 ± 107.1
*C*_max_ (ng/mL)	876.4 ± 249.4	996.6 ± 181.6	95.1 ± 25.9

Collectively, the PK data demonstrate that the i.p. route provides optimal exposure: it achieves rapid absorption, high and sustained systemic concentrations, and good bioavailability, while avoiding the limitations of oral administration. Based on these findings, intraperitoneal injection was selected as the preferred route for subsequent *in vivo* efficacy studies to ensure consistent, therapeutically relevant drug exposure throughout the treatment period.

### 3.7 *In vivo* efficacy of SW-1 in a B16-F10 mouse model

**Figure 6:**
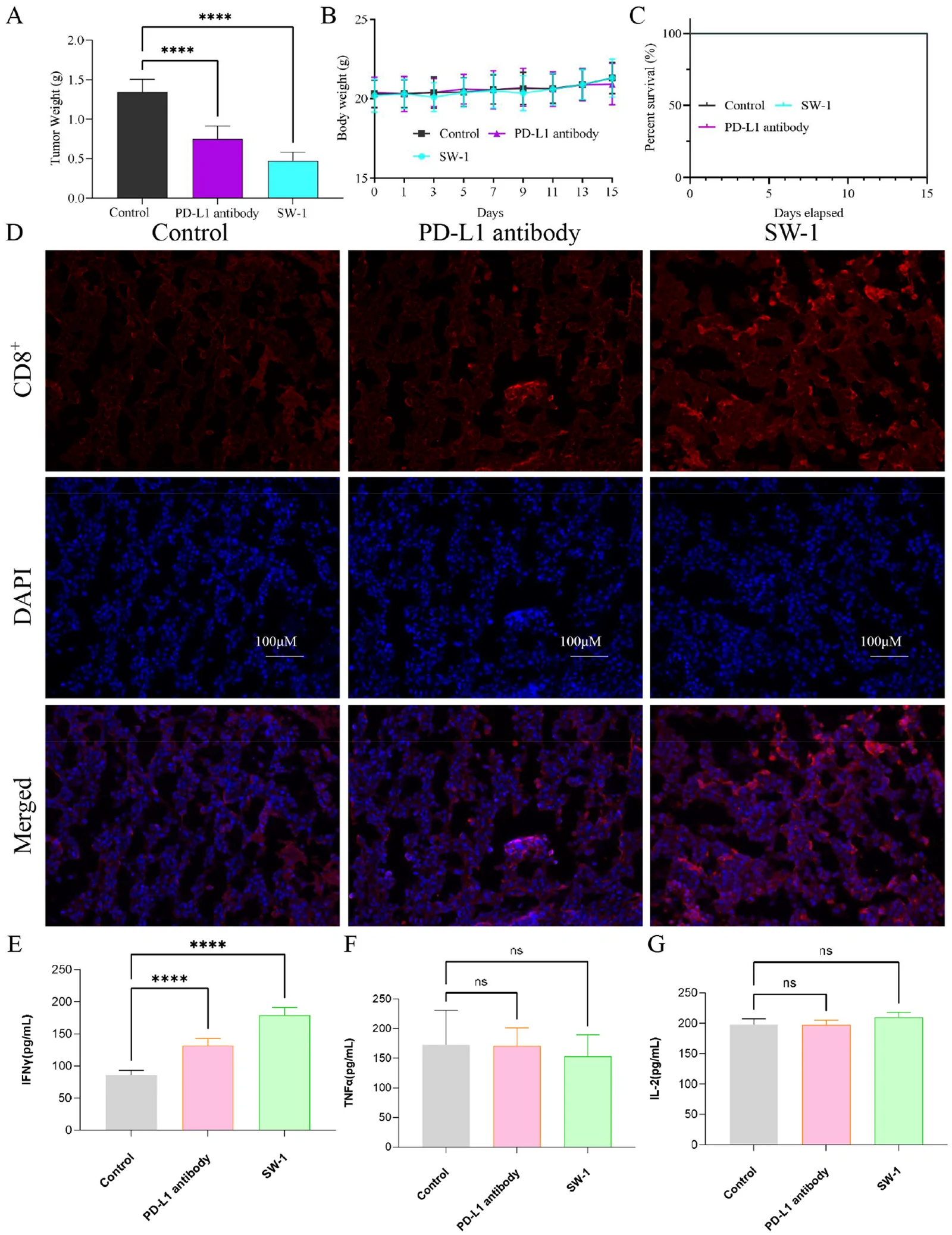
Antitumor effects of SW-1. **(A)** Weights of tumor (n = 6); **(B)** changes of mice body weights (n = 6), day 15, body weights: Control group, 21.3 ± 1.2 g; anti-PD-L1 monotherapy group, 21.3 ± 0.9 g; SW-1 treatment group, 20.9 ± 1.3 g; **(C)** percent survival of mice (n = 6); **(D)** immunofluorescence analysis of CD8+ T cell infiltration in tumor tissues. Quantification of key effector cytokines in tumor homogenates (n = 6): **(E)** The concentrations of IFN-γ in tumor tissues were 85.7 ± 7.3 pg/mL for the Control group, 131.8 ± 11.2 pg/mL for the anti-PD-L1 antibody group, and 178.1 ± 13.5 pg/mL for the SW-1 group, **(F)** The concentrations of TNF-α in tumor tissues were 165.5 ± 40.8 pg/mL for the Control group, 162.0 ± 32.5 pg/mL for the anti-PD-L1 antibody group, and 153.4 ± 35.8 pg/mL for the SW-1 group, and **(G)** The concentrations of IL-2 in tumor tissues were 197.8 ± 9.6 pg/mL for the Control group, 197.6 ± 7.4 pg/mL for the anti-PD-L1 antibody group, and 210.3 ± 7.7 pg/mL for the SW-1 group. All quantitative results are expressed as the mean values with standard deviation (SD). Statistical differences among multiple groups were evaluated using one‑way ANOVA. Statistical significance thresholds were defined as: ns = not significant: P> 0.05, ****P <0.0001.

Encouraged by the potent *in vitro* PD-L1 inhibitory activity and favorable pharmacokinetic profile of SW-1, we further evaluated it’s *in vivo* antitumor efficacy in the B16-F10 melanoma syngeneic mouse model. As shown in **Figure 6A**, both SW-1 and the PD-L1 antibody significantly reduced tumor weight compared to the control group, with SW-1 demonstrating superior efficacy. Specifically, SW-1 exhibited a tumor growth inhibition (TGI) rate of 64.9%, and the PD-L1 antibody group showed a TGI value of 44.2%. Notably, treatment with SW-1 was well tolerated throughout the study, as indicated by stable body weights in all groups (**Figure 6B**) and no overt toxicity. Consistently, the survival curve (**Figure 6C**) showed that SW-1 treatment did not compromise animal survival, further confirming its favorable safety profile. To investigate the immunomodulatory mechanism underlying SW-1’s enhanced antitumor activity, we assessed CD8^+^ T cell infiltration in tumor tissues via immunofluorescence (**Figure 6D**). Compared with both the control and PD-L1 antibody groups, the SW-1-treated group exhibited substantially increased CD8^+^ T cell density within the tumor parenchyma, indicating robust activation of the tumor immune microenvironment. To further validate the functional activation of the immune response, we measured the levels of key pro-inflammatory cytokines in tumor tissues. SW-1 treatment group significantly elevated interferon-γ (IFN-γ) secretion from 85.7 ± 7.3 pg/mL in controls to 178.1 ± 13.5 pg/mL (**Figure 6E**). This increase is consistent with enhanced infiltration of CD8^+^ T cells, as IFN-γ is a hallmark cytokine of CTL effector activity. While tumor necrosis factor-α (TNF-α) and interleukin-2 (IL-2) levels showed a negligible upward trend in treatment groups, these changes did not reach statistical significance (**Figures 6F, G**). This suggests that SW-1 primarily drives IFN-γ production, a critical mediator of anti-tumor immunity, without causing widespread, nonspecific inflammation.

Collectively, these results demonstrate that SW-1 exerts potent antitumor activity *in vivo* while maintaining excellent tolerability. The enhanced therapeutic effect is likely mediated by increased infiltration of CD8^+^ T cells, highlighting SW-1 as a promising immunotherapeutic agent with superior efficacy and safety.

Clinical studies have shown that systemic PD-L1 blockade agents may trigger immune-related cardiac adverse events (irAEs) driven by unrestrained activation of peripheral immune cells, thereby limiting their clinical use. To systematically evaluate the cardiac safety advantage of tumor-targeted SW-1 in mitigating off-tumor immune toxicities, we quantified serum cardiac injury biomarkers, including α-HBDH, AST, LDH, CK-MB, and CK, across three experimental groups. As shown in **Table 3**, the free PD-L1 antibody group exhibited elevated levels of all cardiac enzymes, particularly CK, which reached 961 U/L compared to the control group, indicating potential myocardial inflammatory injury induced by systemic, non-selective checkpoint suppression. In contrast, the SW-1 treatment group maintained cardiac biomarker levels nearly identical to those of the control group, with no significant increases in α-HBDH, AST, LDH, CK-MB, and CK. Collectively, these serum cardiac biochemistry results confirmed that biotin-modified SW-1, with tumor-selective enrichment capability, effectively avoids systemic overactivation of peripheral immune cells, thereby significantly reducing the risk of irAEs compared with untargeted PD-L1 antibody blockade agents.

**Table 3:** Serum cardiac biomarkers (n = 6, Mean ± SD).

Biomarkers	Control	Anti-PD-L1 antibody	SW-1
α-HBDH (U/L)	396 ± 21	422 ± 31	387 ± 26
AST (U/L)	388 ± 33	391 ± 35	386 ± 29
LDH (U/L)	798 ± 40	820 ± 54	774 ± 37
CK-MB (U/L)	193 ± 15	225 ± 19	200 ± 18
CK (U/L)	899 ± 34	961 ± 47	892 ± 32

## 4 Discussion

First-generation immune checkpoint inhibitors, particularly monoclonal antibodies targeting the PD-1/PD-L1 pathway, have revolutionized cancer immunotherapy. However, their clinical efficacy remains severely limited by low objective response rates (typically <20%), primary and acquired resistance, and an inability to adequately activate anti-tumor immunity in “cold” tumor microenvironments. While combinatorial regimens pairing PD-L1 inhibitors with chemotherapy, targeted therapies, or other immunomodulators have shown synergistic activity in clinical trials, co-administration of multiple agents introduces significant challenges, including overlapping toxicities, inconsistent pharmacokinetics, uncoordinated tumor distribution, and unpredictable drug–drug interactions—factors that collectively hinder further clinical translation. Accordingly, rationally designed small-molecule PD-L1 inhibitors represent an attractive alternative, yet most existing candidates suffer from high lipophilicity, poor solubility, and low bioavailability, severely impeding their clinical utility. In this context, biotin-conjugated PD-L1 inhibitor SW-1 emerges as a particularly promising strategy, addressing key limitations of both antibody therapies and conventional small molecules. By leveraging the high expression of the biotin transporter SMVT on many tumor cells, these bifunctional molecules enable tumor-targeted delivery, thereby reducing systemic exposure and off-target effects while enhancing local drug concentration at the tumor site. This targeted approach is particularly critical for PD-L1 inhibitors, as non-specific binding to immune cells or healthy tissues can lead to irAEs and diminished therapeutic efficacy.

To directly verify that SW-1 reshapes an immunologically favorable tumor microenvironment by relieving PD-L1-mediated immune suppression, we characterized intratumoral CD8^+^ T cell infiltration and effector cytokine secretion, two core markers of robust anti-tumor cytotoxic immunity. Immunofluorescence staining confirmed markedly elevated CD8^+^ T cell abundance within the tumor parenchyma after SW-1 monotherapy, relative to the vehicle control and single PD-L1 antibody groups, providing direct histological evidence of T cell recruitment into previously immune-excluded cold tumors. Consistent with this, ELISA quantification of intratumoral effector cytokines further supported the immune-activating capacity of SW-1: SW-1 treatment induced a prominent elevation of intratumoral IFN-γ from 85.7 ± 7.3 pg/mL in control tumors to 178.1 ± 13.5 pg/mL. As the signature functional cytokine secreted by activated cytotoxic CD8^+^ T lymphocytes, robust upregulation of IFN-γ aligns with increased tumor-infiltrating CD8^+^ T cells, demonstrating that SW-1 effectively restores the effector function of tumor-resident CTLs. In contrast, intratumoral TNF-α and IL-2 levels showed only mild, statistically nonsignificant increases upon SW-1 administration. This selective induction of IFN-γ indicates that SW-1 triggers specific anti-tumor cytotoxic immune responses rather than broad, nonspecific inflammatory activation, thereby helping avoid systemic inflammatory side effects.

Additionally, it is important to clarify that SMVT-mediated tumor enrichment of SW-1 will not impair its intratumor PD-L1-blocking function. After entering tumor cells via the biotin-SMVT pathway, residual free SW-1 persists in the tumor interstitial fluid. As a small molecule targeting the extracellular domain of PD-L1, free SW-1 can bind PD-L1 on the surface of infiltrating immune cells without transporter-mediated uptake, thereby efficiently disrupting PD-1/PD-L1 interactions between immune and tumor cells. The elevated CD8^+^ T-cell infiltration and subsequent IFN-γ production in treated tumor tissues jointly validate this functional effect. Compared with non-targeted PD-L1 inhibitors, SW-1 concentrates in tumors and has low systemic exposure in immune organs, thereby balancing potent intratumor immune activation with a lower risk of immune-related adverse reactions.

Moreover, the biotin moiety not only serves as a targeting ligand but also improves the molecule’s overall physicochemical properties, including solubility and pharmacokinetic behavior, thereby addressing common drawbacks of small-molecule PD-L1 inhibitors. Unlike combination therapies, a single biotin-conjugated PD-L1 inhibitor offers unified and predictable pharmacokinetics, synchronized tumor accumulation, and enhanced tissue penetration, thereby enabling more consistent and effective blockade of the PD-1/PD-L1 axis. By selectively enriching in tumor tissues, these agents can more effectively reverse PD-L1-mediated immune suppression, promote CD8^+^ T-cell infiltration, and convert “cold” tumors into immunologically “hot” ones—ultimately enhancing anti-tumor immunity while minimizing systemic toxicity. In summary, the development of biotin-conjugated PD-L1 inhibitors, represented by SW-1, is a rational and powerful strategy to overcome the limitations of current PD-L1-targeted therapies. By combining the advantages of small-molecule inhibitors with tumor-specific delivery, these agents have the potential to improve therapeutic efficacy, reduce adverse events, and expand the population of patients who benefit from cancer immunotherapy.

## 5 Conclusions

Herein, we rationally designed SW-1, a biotin-conjugated small-molecule PD-L1 inhibitor, using CADD strategies. SW-1 potently blocks the PD-1/PD-L1 interaction with an IC_50_ of 5.6 ± 0.3 nM and binds human and murine PD-L1 with high affinity. In B16-F10 syngeneic melanoma mice, SW-1 achieves a 64.9% inhibition of tumor growth without systemic toxicity, far outperforming PD-L1 antibody monotherapy. Mechanistically, SW-1 accumulates in tumors via SMVT-mediated biotin transport, elevating intratumoral CD8^+^ T-cell infiltration and IFN-γ secretion, thereby remodeling immunosuppressive cold tumors. This bifunctional scaffold overcomes the major drawbacks of antibody and conventional small-molecule PD-L1 agents. SW-1 serves as a high-potency, tumor-selective lead candidate for subsequent preclinical optimization of targeted tumor immunotherapy.

## Data Availability

The original contributions presented in the study are included in the article/**Supplementary Material**, further inquiries can be directed to the corresponding author/s.

## Supplementary material

The Supplementary Material for this article can be found online https://www.frontiersin.org/articles/10.3389/fimmu.2026.1879839/full#supplementary-material
