# Supplementary material for: Rational design of D(+)-biotin-conjugated resorcinol dibenzyl ethers as tumor-targeted PD-L1 inhibitors for precision cancer immunotherapy
Source: Front Immunol. 2026 Sep 3;17:1879839. doi: 10.3389/fimmu.2026.1879839 (PMC13582261; doi:10.3389/fimmu.2026.1879839)
Supplement: Supplementary file 1 [file DataSheet1.pdf]

Supporting Information

Rational Design of D(+)-Biotin-Conjugated Resorcinol Diben-zyl Ethers as Tumor-Targeted PD-L1 Inhibitors for Precision Cancer Immunotherapy

Table of Contents

Copies of SW1-7 ^{1}H NMR and ^{13}C NMR spectra ……………………………………………………………………S2

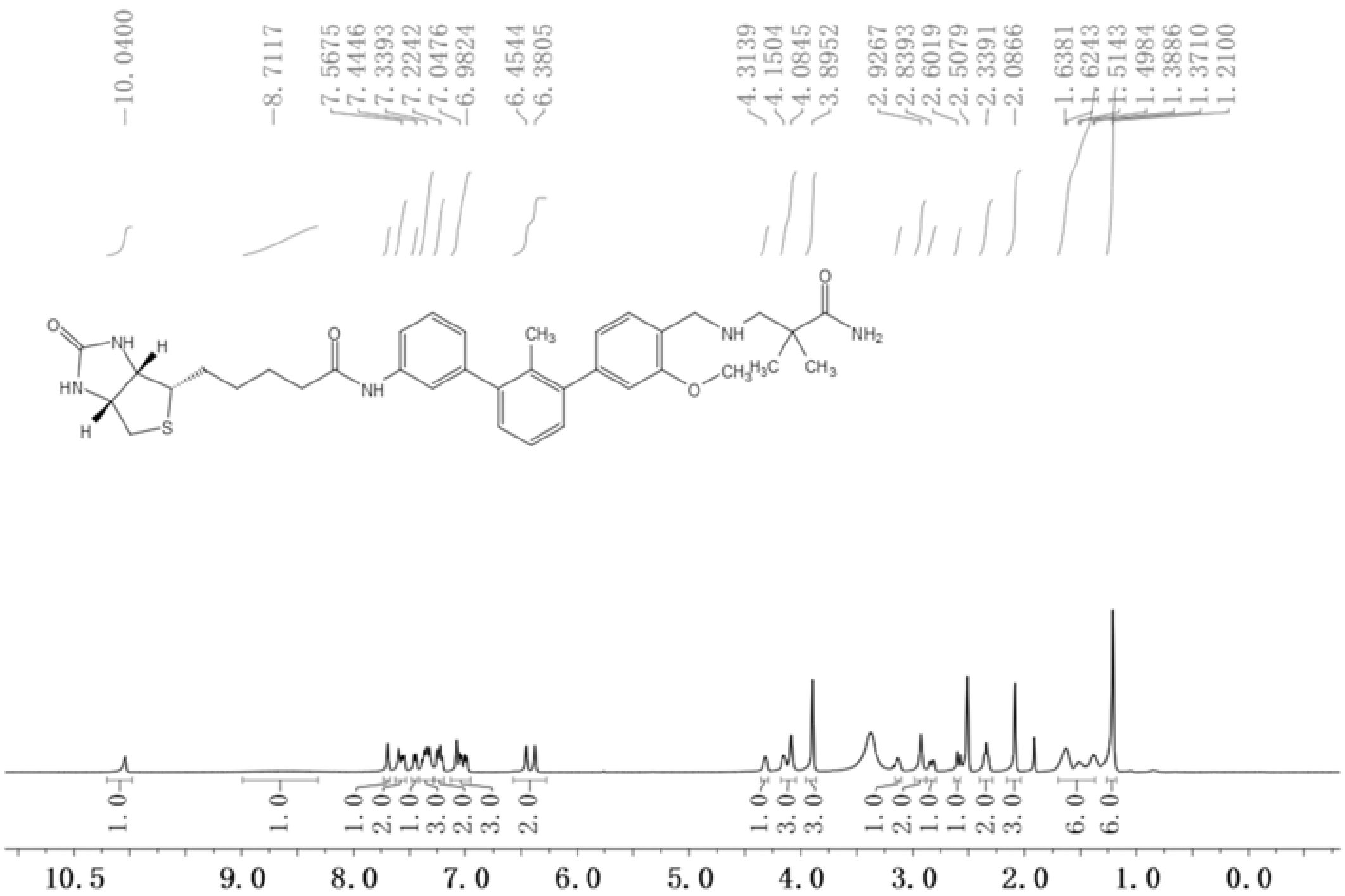
10.0400
8.7117
7.5675
7.4446
7.3393
7.2242
7.0476
6.9824
6.4544
6.3805
4.3139
4.1504
4.0845
3.8952
2.9267
2.8393
2.6019
2.5079
2.3391
2.0866
1.6381
1.6243
1.5143
1.4984
1.3886
1.3710
1.2100
O
NH
H
HN
H
S
O
NH
CH3
NH
O
NH2
O
CH3
H3C
CH3
1.0
1.0
1.0
2.0
1.0
3.0
2.0
3.0
2.0
1.0
3.0
3.0
1.0
2.0
1.0
1.0
2.0
3.0
6.0
6.0
10.5
9.0
8.0
7.0
6.0
5.0
4.0
3.0
2.0
1.0
0.0

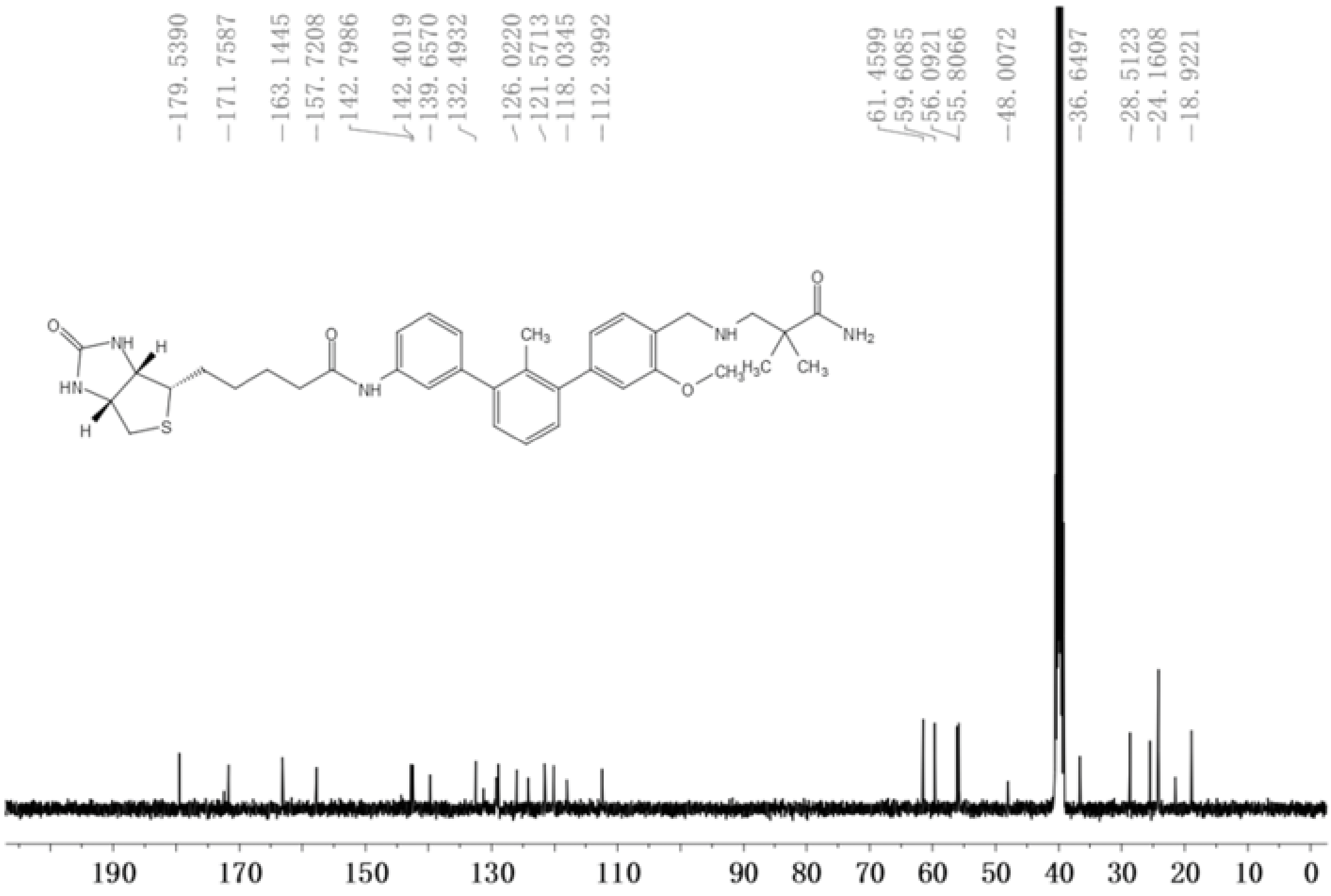
179.5390
171.7587
163.1445
157.7208
142.7986
142.4019
139.6570
132.4932
126.0220
121.5713
118.0345
112.3992
61.4599
59.6085
56.0921
55.8066
48.0072
36.6497
28.5123
24.1608
18.9221
O
NH
H
HN
H
S
O
NH
CH3
NH
O
NH2
O
CH3
H3C
CH3
190
170
150
130
110
90
80
70
60
50
40
30
20
10
0

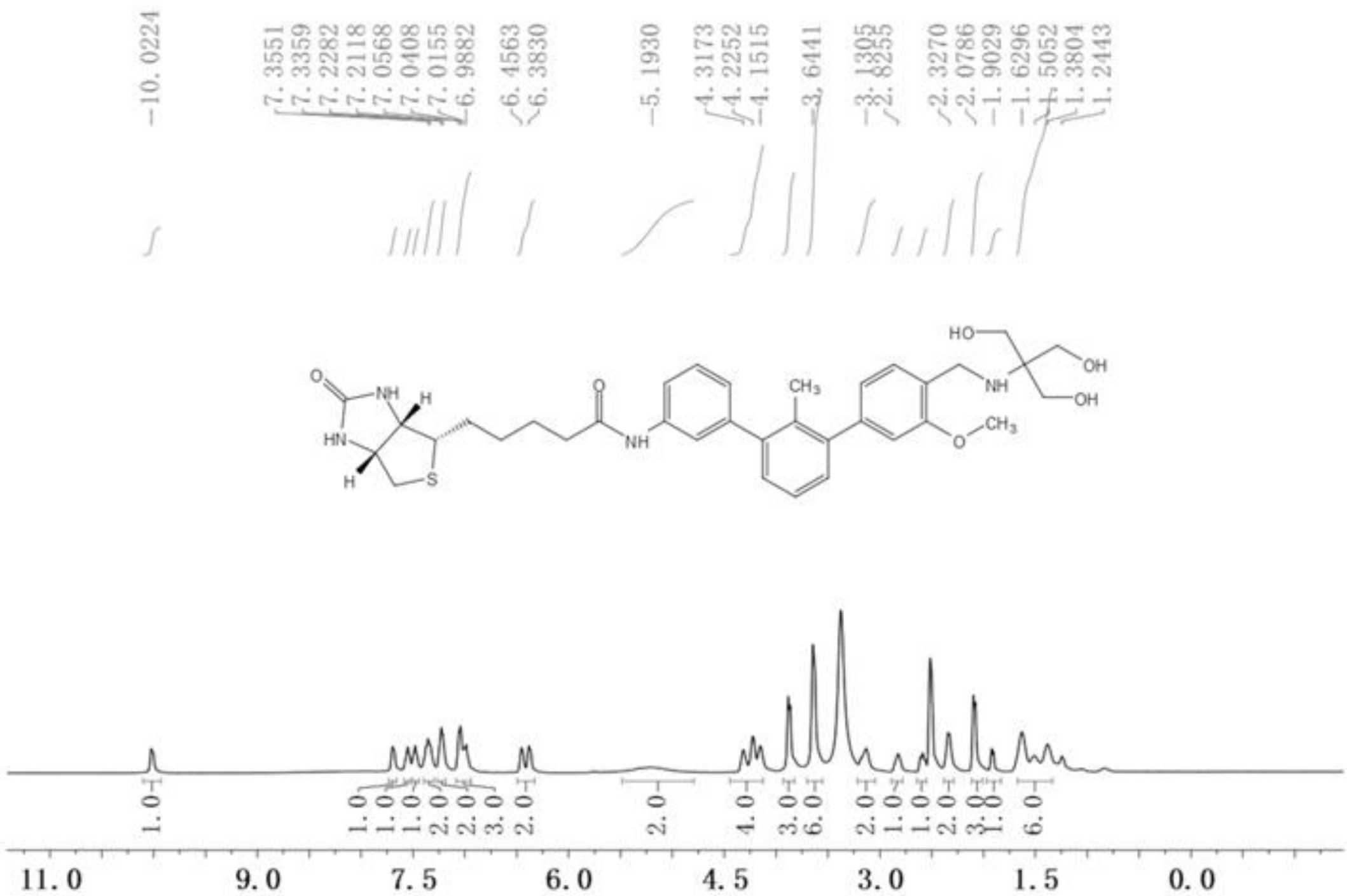
10.0224
7.3551
7.3359
7.2282
7.2118
7.0568
7.0408
7.0155
6.9882
6.4563
6.3830
5.1930
4.3173
4.2252
4.1515
3.6441
3.1305
2.8255
2.3270
2.0786
1.9029
1.6296
1.5052
1.3804
1.2443
HO
OH
OH
NH
CH3
O
NH
HN
H
S
O
CH3
1.0
1.0
1.0
1.0
2.0
2.0
3.0
2.0
2.0
4.0
3.0
6.0
2.0
1.0
1.0
2.0
3.0
1.0
6.0
11.0
9.0
7.5
6.0
4.5
3.0
1.5
0.0

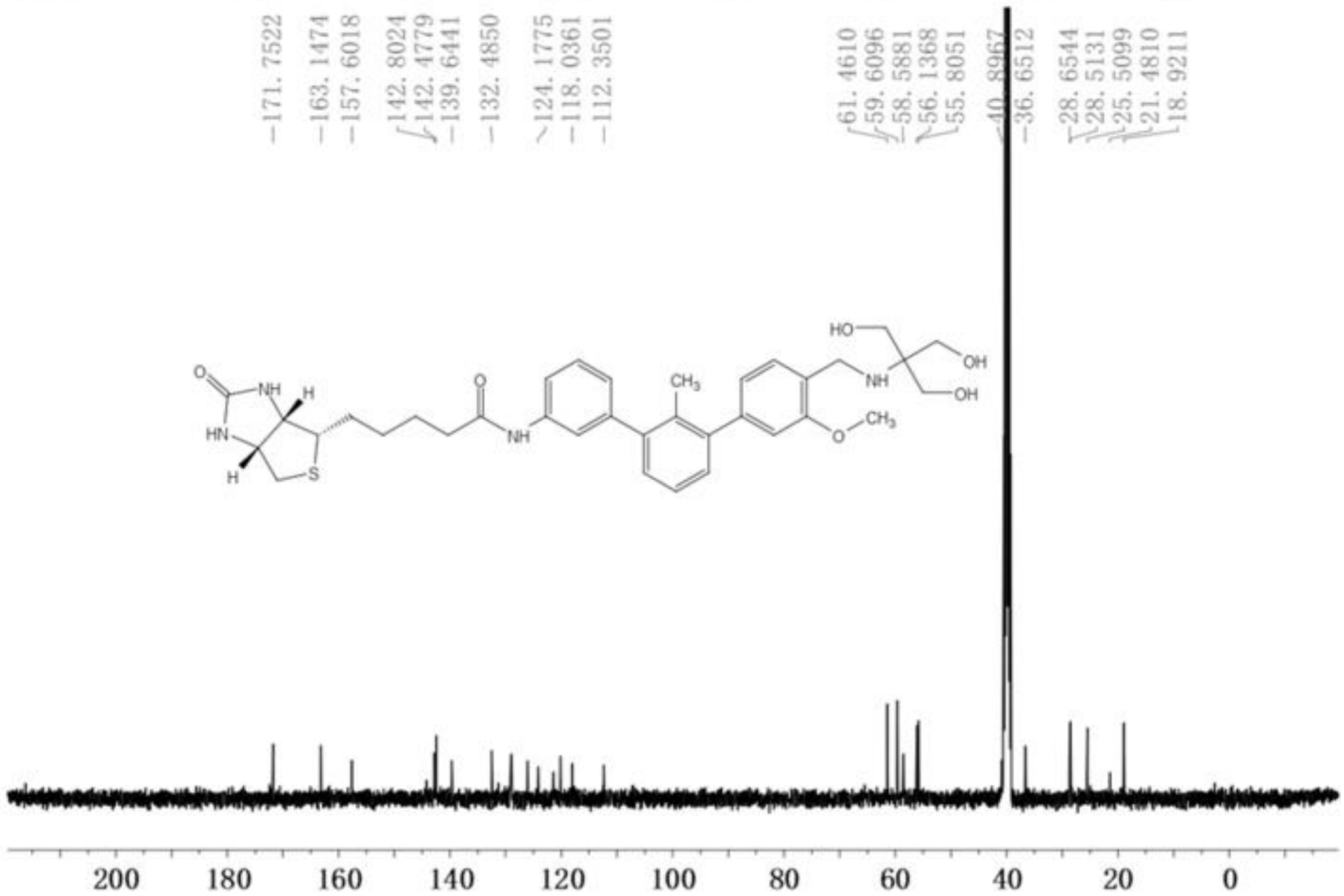
171.7522
163.1474
157.6018
142.8024
142.4779
139.6441
132.4850
124.1775
118.0361
112.3501
61.4610
59.6096
58.5881
56.1368
55.8051
40.8967
36.6512
28.6544
28.5131
25.5099
21.4810
18.9211
HO
OH
OH
NH
CH3
O
NH
HN
H
S
O
CH3
200
180
160
140
120
100
80
60
40
20
0

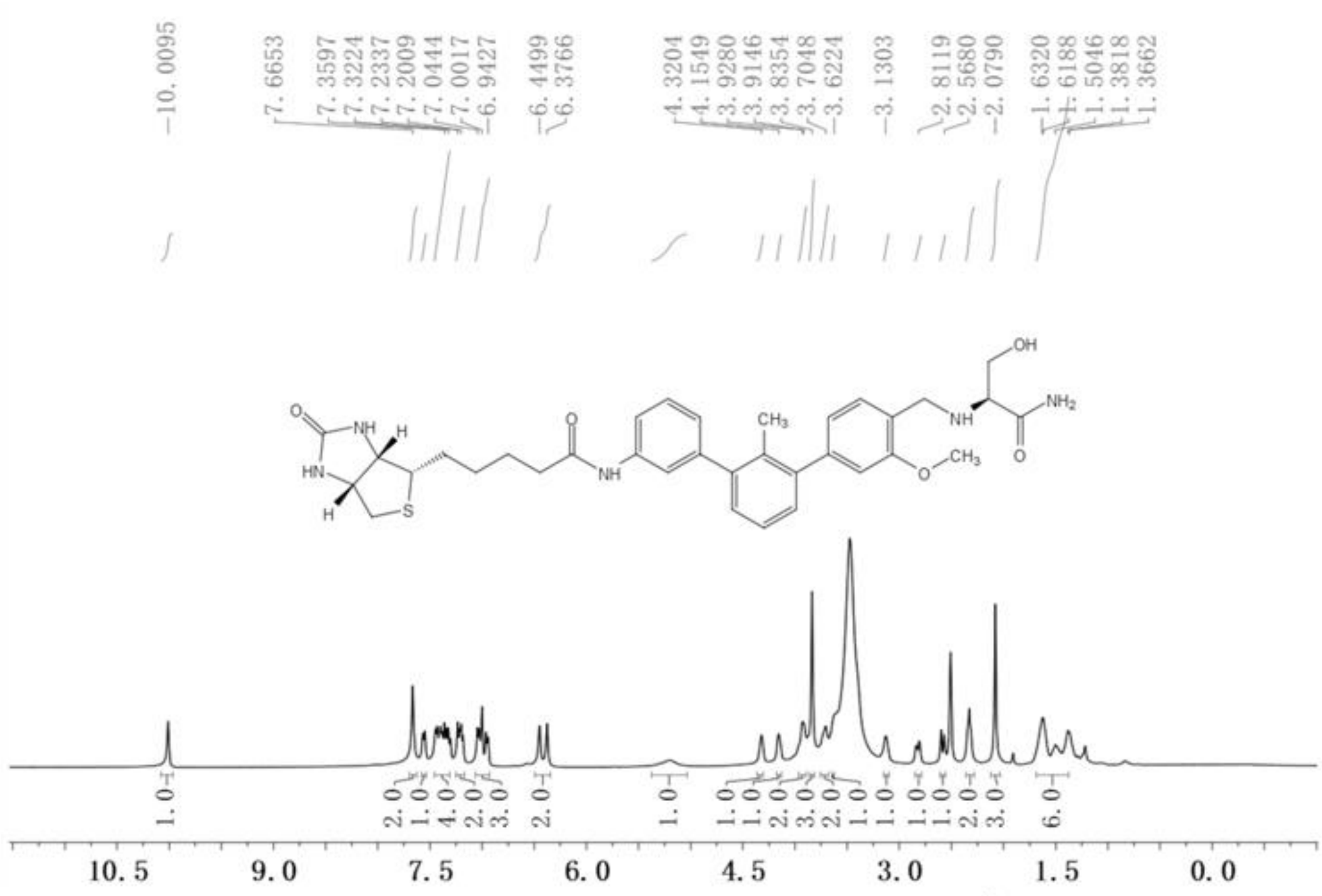
10.0095
7.6653
7.3597
7.3224
7.2337
7.2009
7.0444
7.0017
6.9427
6.4499
6.3766
4.3204
4.1549
3.9280
3.9146
3.8354
3.7048
3.6224
3.1303
2.8119
2.5680
2.0790
1.6320
1.6188
1.5046
1.3818
1.3662
OH
O
NH
H
HN
H
S
NH
CH3
NH
NH2
O
O
CH3
1.0
2.0
1.0
4.0
2.0
3.0
2.0
1.0
1.0
1.0
2.0
3.0
2.0
1.0
1.0
1.0
1.0
2.0
3.0
6.0
10.5
9.0
7.5
6.0
4.5
3.0
1.5
0.0

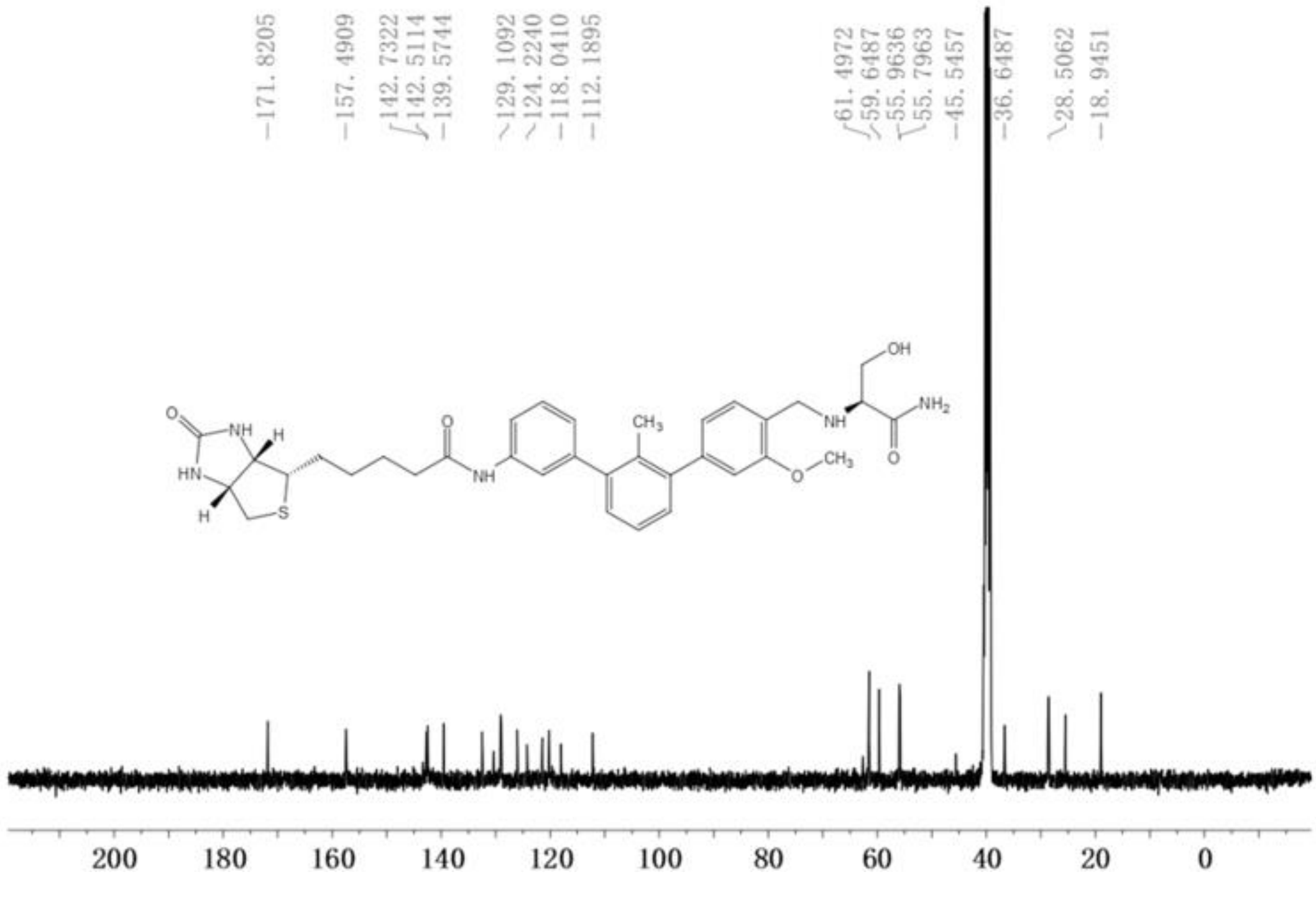
171.8205
157.4909
142.7322
142.5114
139.5744
129.1092
124.2240
118.0410
112.1895
61.4972
59.6487
55.9636
55.7963
45.5457
36.6487
28.5062
18.9451
OH
O
NH
H
HN
H
S
NH
CH3
NH
NH2
O
O
CH3
200
180
160
140
120
100
80
60
40
20
0

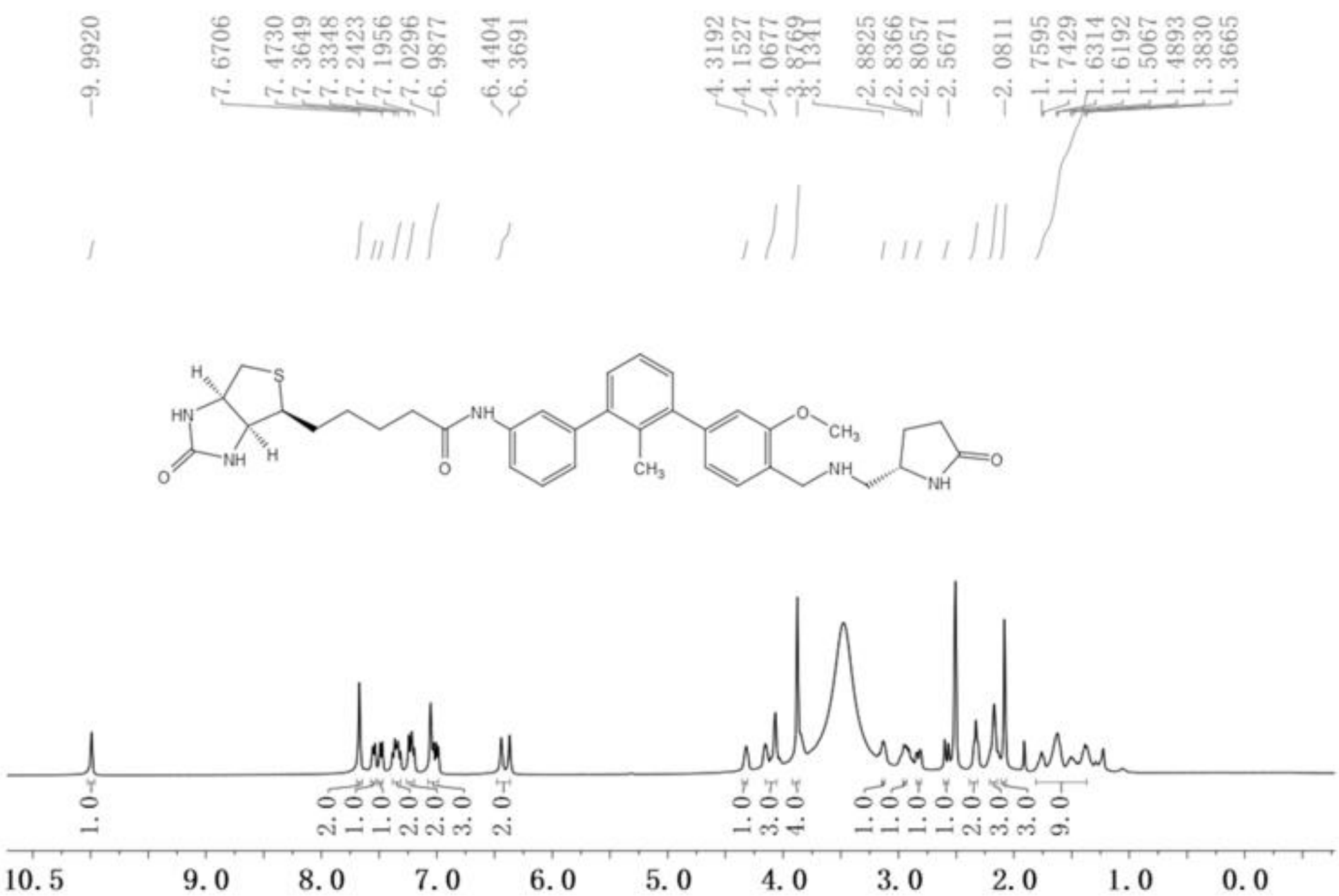
9.9920
7.6706 7.4730 7.3649 7.3348 7.2423 7.1956 7.0296 6.9877
6.4404 6.3691
4.3192 4.1527 4.0677 3.8769 3.1341
2.8825 2.8366 2.8057 2.5671
2.0811 1.7595 1.7429 1.6314 1.6192 1.5067 1.4893 1.3830 1.3665
CH3
NH
HN
H
S
O
1.0 2.0 1.0 1.0 2.0 2.0 3.0 2.0 1.0 3.0 4.0 1.0 1.0 1.0 1.0 2.0 3.0 3.0 9.0
10.5 9.0 8.0 7.0 6.0 5.0 4.0 3.0 2.0 1.0 0.0

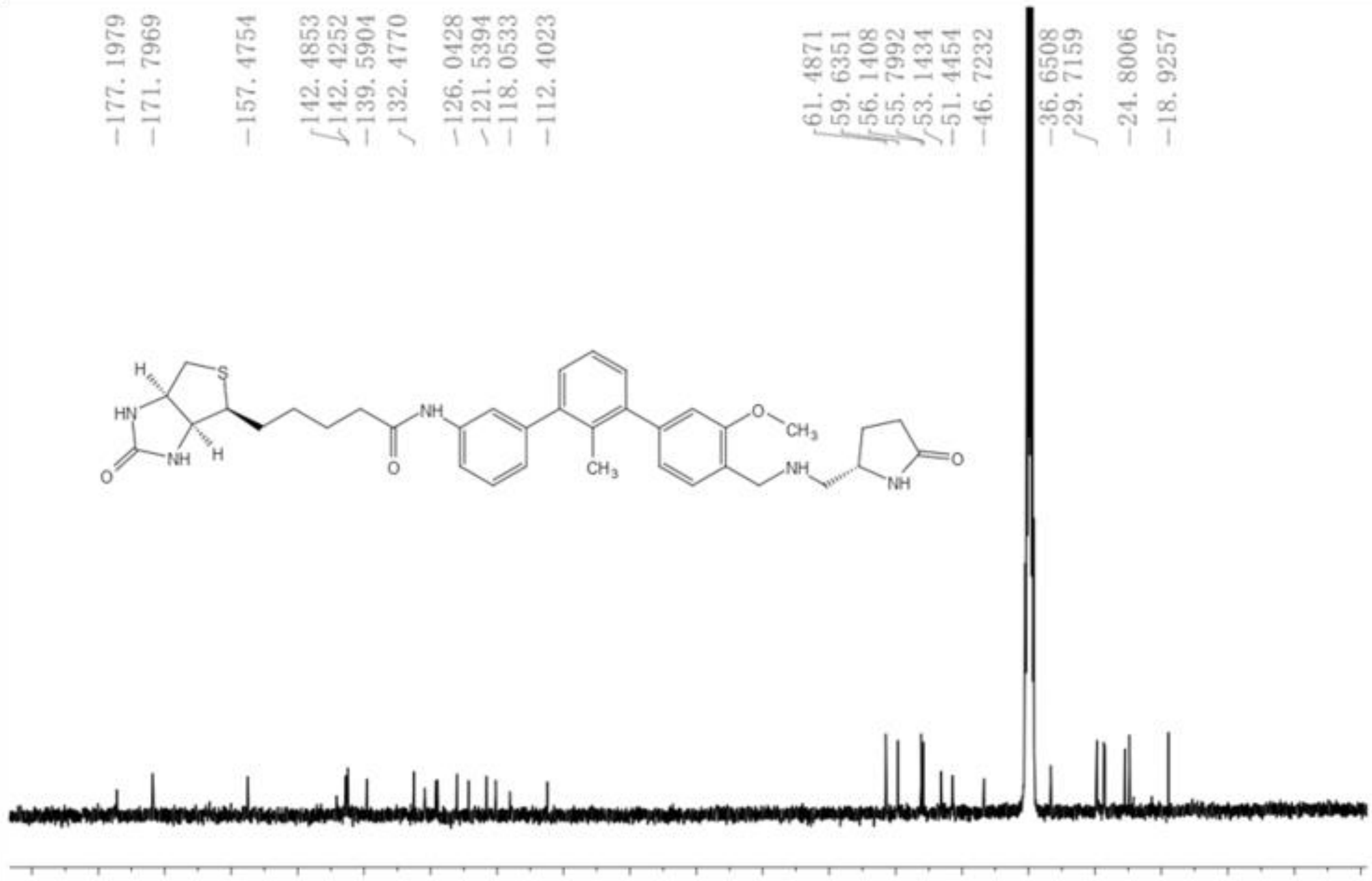
177.1979 171.7969
157.4754
142.4853 142.4252 139.5904 132.4770
126.0428 121.5394 118.0533 112.4023
61.4871 59.6351 56.1408 55.7992 53.1434 51.4454 46.7232
36.6508 29.7159 24.8006 18.9257
CH3
NH
HN
H
S
O
190 170 150 130 110 90 80 70 60 50 40 30 20 10 0

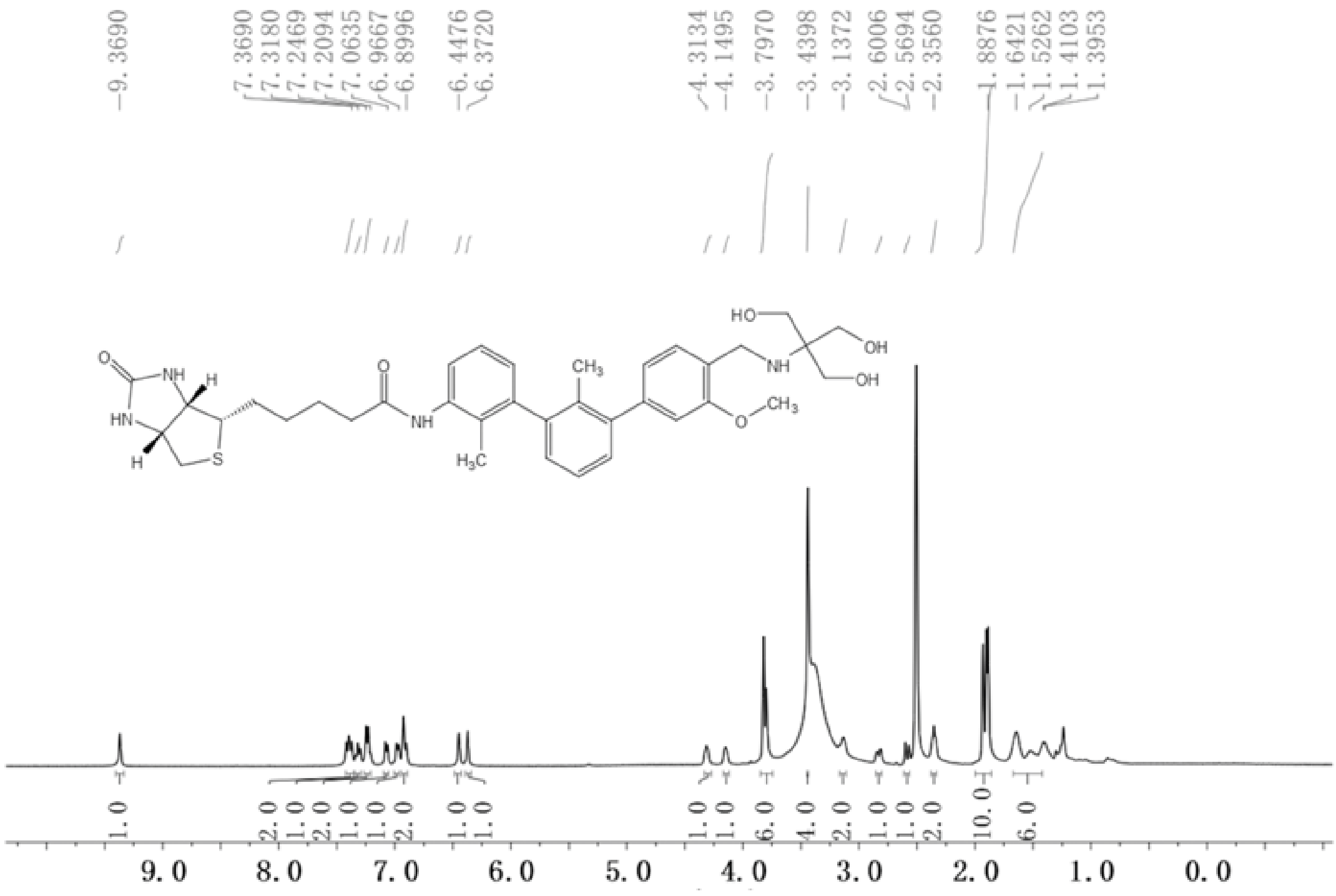
9.3690
7.3690
7.3180
7.2469
7.2094
7.0635
6.9667
6.8996
6.4476
6.3720
4.3134
4.1495
3.7970
3.4398
3.1372
2.6006
2.5694
2.3560
1.8876
1.6421
1.5262
1.4103
1.3953
1.0
2.0
1.0
2.0
1.0
2.0
1.0
1.0
1.0
1.0
6.0
4.0
2.0
1.0
2.0
10.0
6.0
9.0
8.0
7.0
6.0
5.0
4.0
3.0
2.0
1.0
0.0

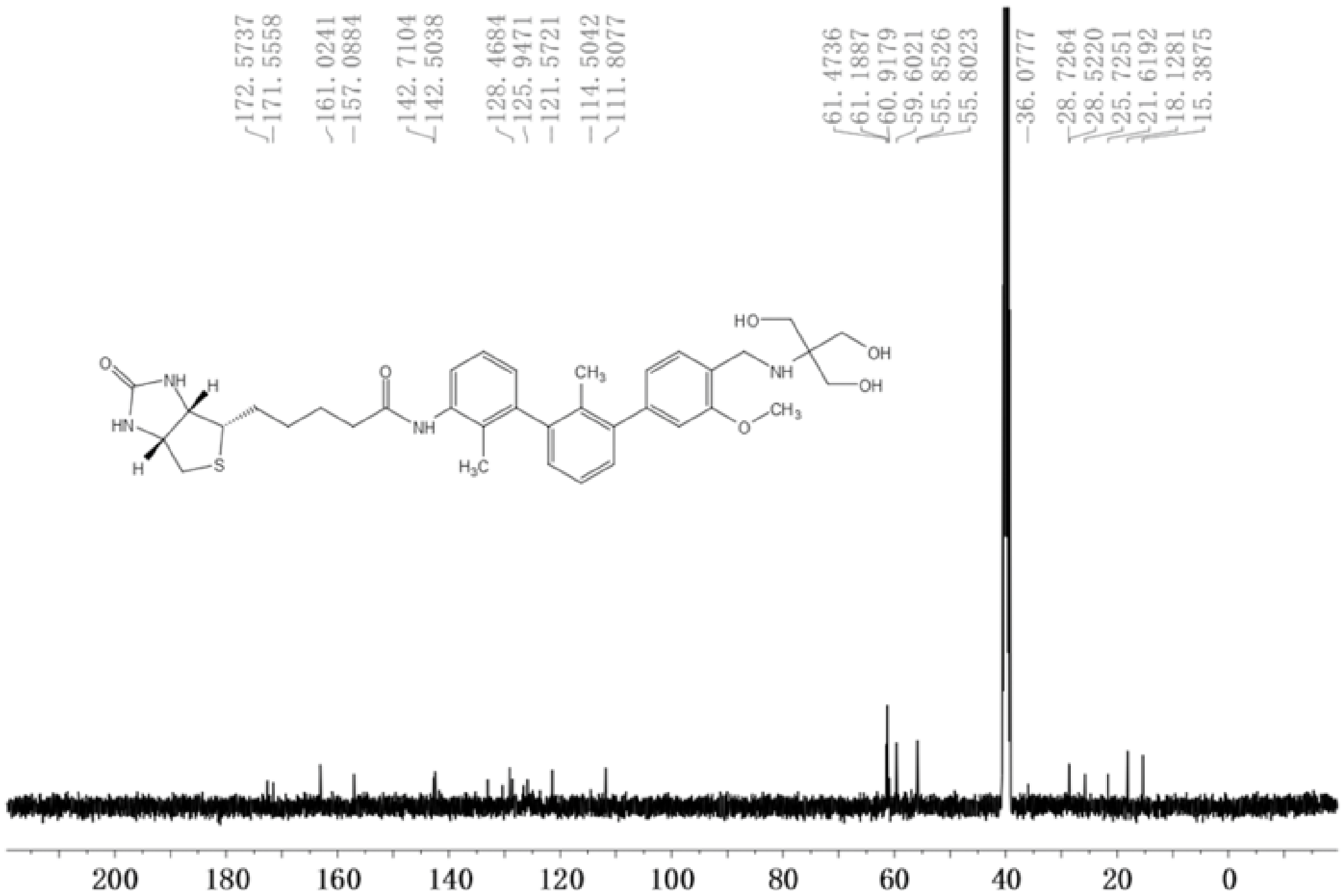
172.5737
171.5558
161.0241
157.0884
142.7104
142.5038
128.4684
125.9471
121.5721
114.5042
111.8077
61.4736
61.1887
60.9179
59.6021
55.8526
55.8023
36.0777
28.7264
28.5220
25.7251
21.6192
18.1281
15.3875
200
180
160
140
120
100
80
60
40
20
0

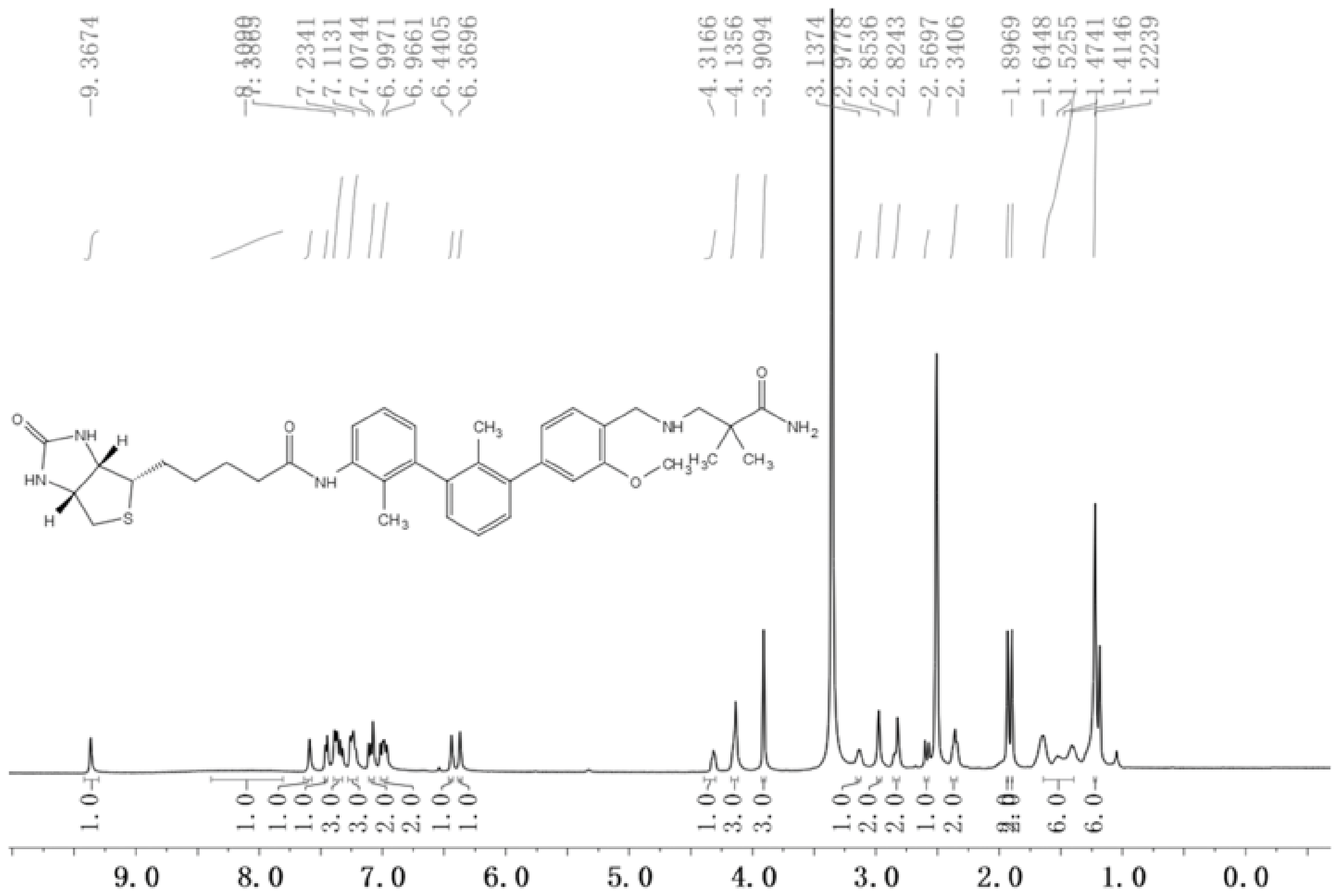
9.3674
7.2341
7.1131
7.0744
6.9971
6.9661
6.4405
6.3696
4.3166
4.1356
3.9094
3.1374
2.9778
2.8536
2.8243
2.5697
2.3406
1.8969
1.6448
1.5255
1.4741
1.4146
1.2239
9.0 8.0 7.0 6.0 5.0 4.0 3.0 2.0 1.0 0.0

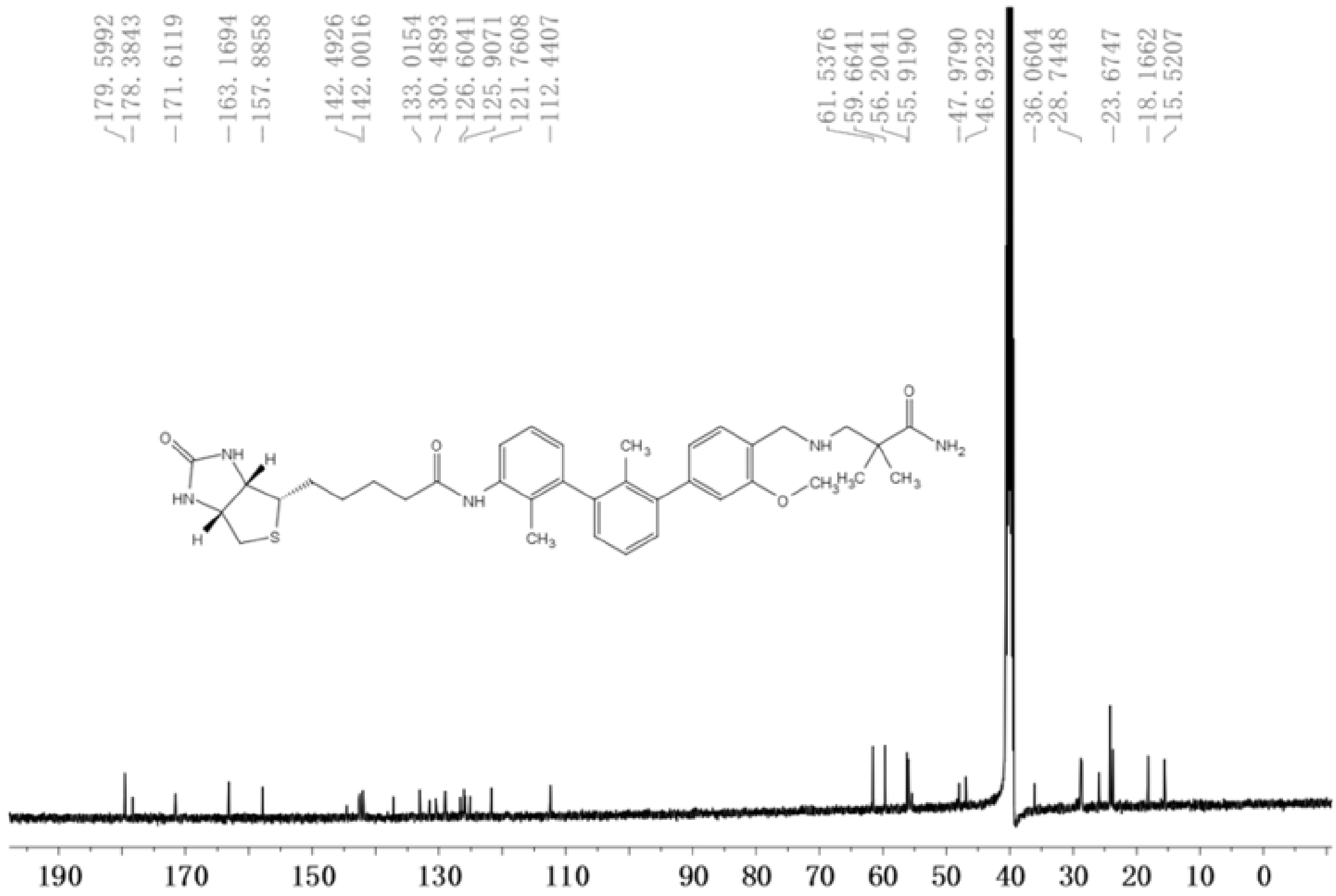
179.5992
178.3843
171.6119
163.1694
157.8858
142.4926
142.0016
133.0154
130.4893
126.6041
125.9071
121.7608
112.4407
61.5376
59.6641
56.2041
55.9190
47.9790
46.9232
36.0604
28.7448
23.6747
18.1662
15.5207
190 170 150 130 110 90 80 70 60 50 40 30 20 10 0

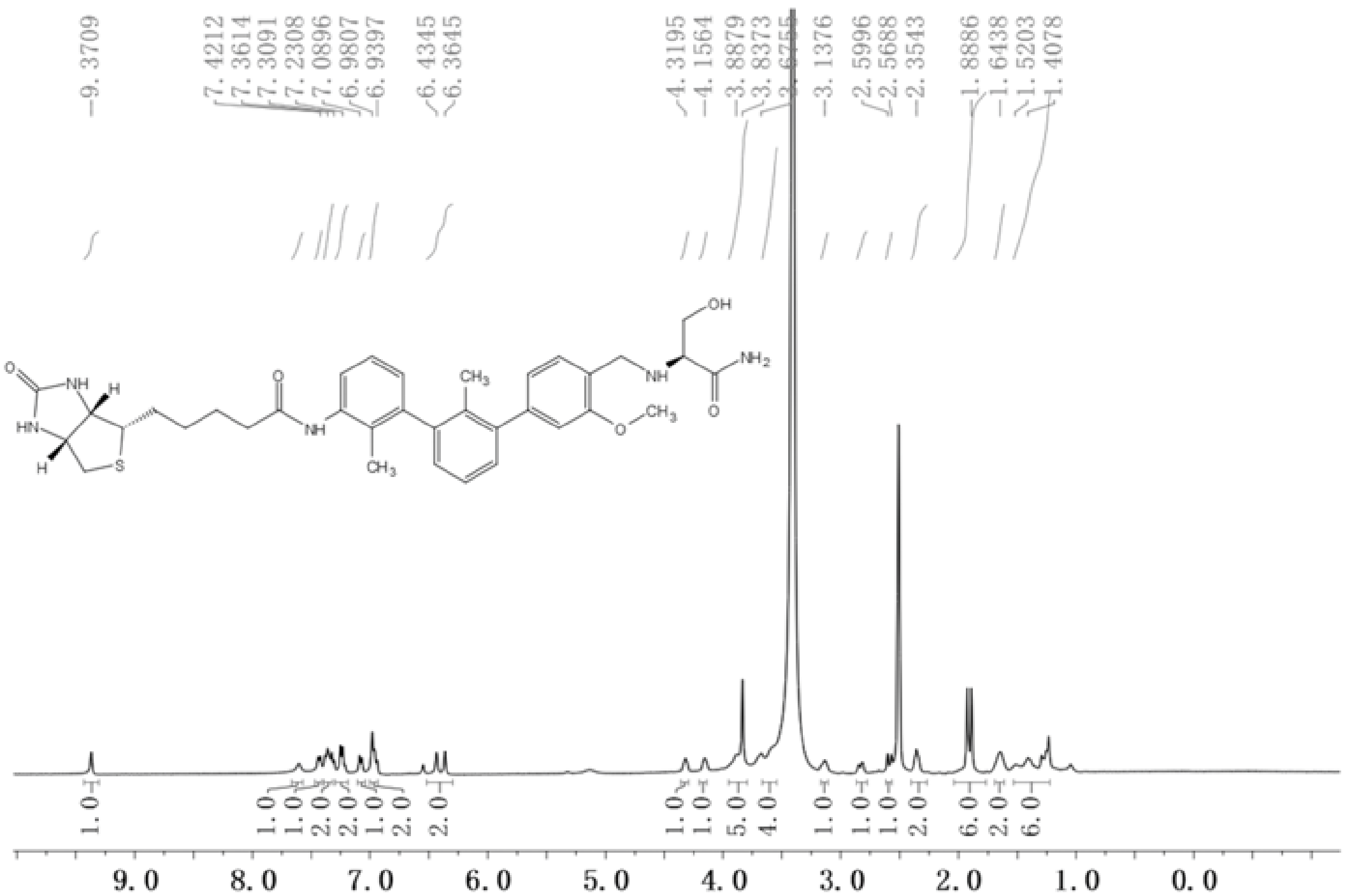
9.3709
7.4212
7.3614
7.3091
7.2308
7.0896
6.9807
6.9397
6.4345
6.3645
4.3195
4.1564
3.8879
3.8373
3.1376
2.5996
2.5688
2.3543
1.8886
1.6438
1.5203
1.4078
9.0
8.0
7.0
6.0
5.0
4.0
3.0
2.0
1.0
0.0

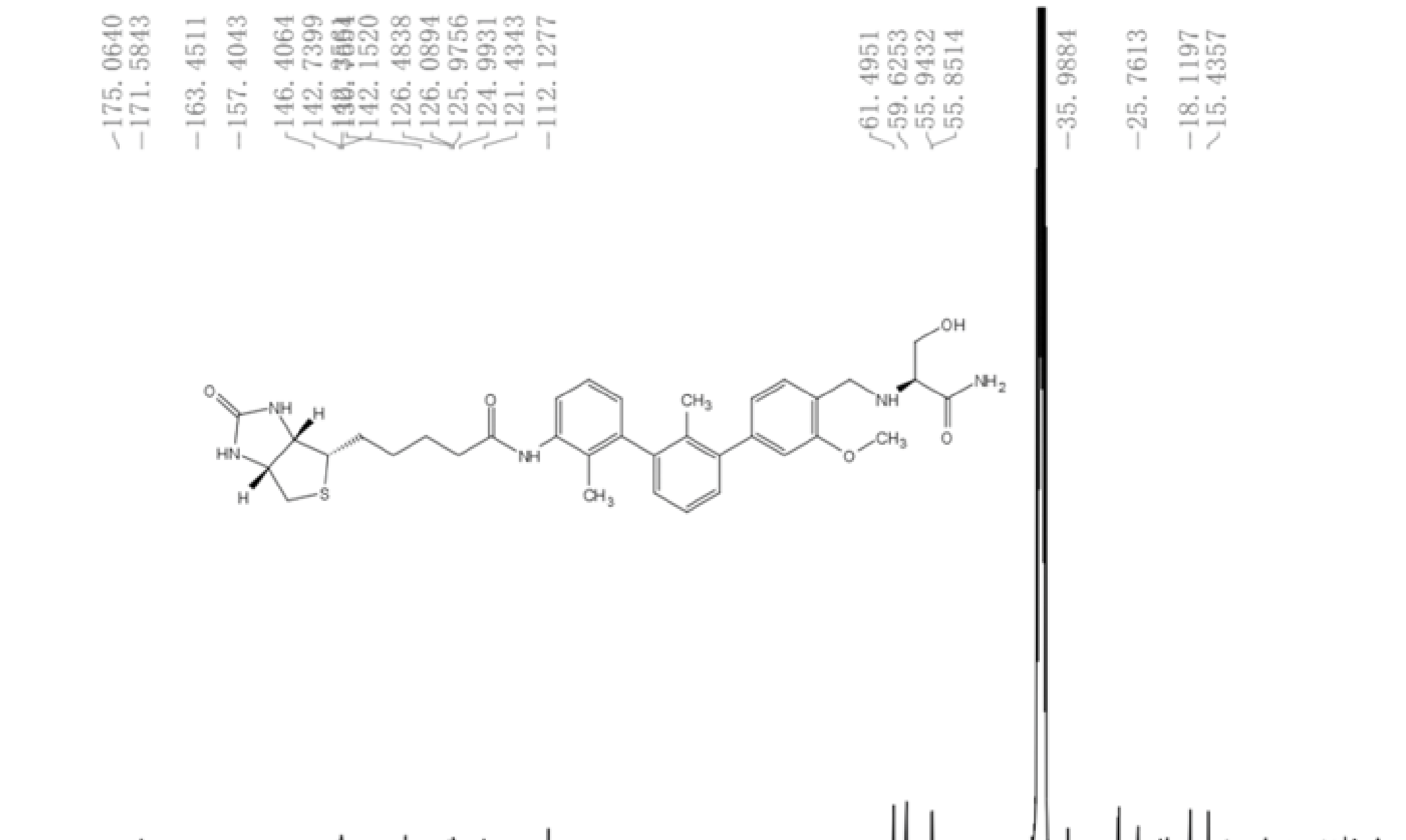
175.0640
171.5843
163.4511
157.4043
146.4064
142.7399
142.1520
126.4838
126.0894
125.9756
124.9931
121.4343
112.1277
61.4951
59.6253
55.9432
55.8514
35.9884
25.7613
18.1197
15.4357
170
150
130
110
90
80
70
60
50
40
30
20
10
0
